# Chaotic Lévy and adaptive restart enhance the Manta Ray foraging optimizer for gene feature selection

**DOI:** 10.1038/s41598-025-25766-y

**Published:** 2025-11-25

**Authors:** Shamsuddeen Adamu, Hitham Alhussian, Said Jadid Abdulkadir, Ayed Alwadain, Sallam O. F. Khairy, Hussaini Mamman, Ismail Said Almuniri, Al Waleed Sulaiman Al Abri, Zaid Fawaz Jarallah, Hamood Saif Hamood Al Fahdi, Maged Nasser, Bander Ali Saleh Al-Rimy

**Affiliations:** 1 Department of Computing, Universiti Teknologi PETRONAS, Seri Iskandar, Malaysia; 2 IAIICT, Ahmadu Bello University, Zaria, Nigeria; 3 Computer Science Department, Community College, King Saud University, Riyadh, Saudi Arabia; 4 Department of Information Systems, College of Economics, Management and Information Systems, University of Nizwa, Nizwa, Oman; 5 Computer Science Department, College of Education for Pure Science, University of Mosul, Mosul, Iraq; 6 Computer Science Department, Aljabel Alakhder Sultanate of Oman, Muscat, Oman; 7 School of Computing, University of Portsmouth, Portsmouth, UK England

**Keywords:** Metaheuristic optimization, Manta Ray foraging optimization, Feature selection, Lévy flight and chaotic maps, Gene expression classification, Multi-objective optimization, Hybrid optimization algorithms, Bioinformatics, Cancer, Computational biology and bioinformatics, Mathematics and computing

## Abstract

Swarm-based optimization algorithms often face challenges in maintaining an effective exploration–exploitation balance in high-dimensional search spaces. Manta Ray Foraging Optimization (MRFO), while competitive, is hindered by static parameter settings and premature convergence. This study introduces CLA-MRFO, an adaptive variant incorporating chaotic Lévy flight modulation, phase-aware memory, and an entropy-informed restart strategy to enhance search dynamics. On the CEC’17 benchmark suite, CLA-MRFO achieved the lowest mean error on 23 of 29 functions, with an average performance gain of 31.7% over the next best algorithm; statistical validation via the Friedman test confirmed the significance of these results ($p < 0.01$). To examine practical utility, CLA-MRFO was applied to a high-dimensional leukemia gene selection task, where it identified ultra-compact subsets ($\le$5% of original features) of biologically coherent genes with established roles in leukemia pathogenesis. These subsets enabled a mean F_1_-score of $0.953 \pm 0.012$ under a stringent 5-fold nested cross-validation across six classification models. While highly effective in a binary classification setting, the method’s performance in a multi-class diagnostic context revealed constraints in generalizability, indicating that the identified biomarkers are highly context-dependent. Overall, CLA-MRFO exhibited consistent behavior (<5% variance across runs) and provides an adaptable framework for high-dimensional optimization tasks with applications extending to bioinformatics and related domains.

## Introduction

Modern optimization problems across diverse domains, from engineering design to machine learning (ML) and bioinformatics, frequently contend with high-dimensional, multimodal, and deceptive search landscapes^1,2^. A quintessential example lies in biomedical informatics, such as gene expression-based leukemia classification, where microarray technologies yield thousands of features per sample. In these scenarios, identifying truly informative subsets of features amidst vast quantities of redundant or noisy data becomes a paramount, yet intensely challenging, optimization task due to the inherent “curse of dimensionality” and the presence of numerous local optima^3^.

To address such complexities, swarm intelligence (SI) algorithms, which draw inspiration from the collective behaviors observed in natural systems, have emerged as a powerful class of metaheuristics^4–6^. SI methods are highly regarded for their intrinsic ability to balance global exploration of the search space with targeted local exploitation of promising regions, making them highly attractive for tackling real-world problems including gene selection, neural architecture design, and complex scheduling.

Significant research efforts have focused on enhancing the adaptability and robustness of SI algorithms. For example, chaotic dynamics have been effectively integrated into Particle Swarm Optimization (PSO) to counteract stagnation and improve exploration^7–10^, while memory mechanisms in Differential Evolution (DE) aid in preserving high-quality solutions across generations^11–13^. However, despite MRFO’s acknowledged potential^14^, analogous adaptive enhancements specifically designed to address its core weaknesses remain relatively under explored. MRFO consistently exhibits vulnerabilities on the more complex CEC’17 functions (f10–f30), where no single existing approach reliably achieves the necessary balance of precision and robustness across diverse function types^15^. Addressing this critical gap is paramount, as numerous practical applications—including optimizing neural network architectures where premature convergence leads to suboptimal models^16^, or streamlining complex supply chain logistics^17,18^—increasingly resemble the multimodal and hybrid complexity of these benchmarks, demanding more versatile and reliable optimization tools.

To overcome these documented limitations, this paper proposes the Synergistic Chaotic Lévy and Adaptive Restart Variant of MRFO (CLA-MRFO). The core contributions of this work are as follows:

1. **Chaotic Lévy flight (LF) Modulation:** Dynamic step-size adjustment using chaotic maps within LF to invigorate global exploration and counteract premature convergence.
2. **Phase-Specific Memory Banks:** Preservation of diverse elite solutions across foraging modes.
3. **Entropy-Informed Adaptive Restart:** Stagnation-triggered mechanism that injects diversity into the population without undermining convergence stability.

These components work in synergy to enable adaptive transitions between exploration and exploitation, systematically mitigating MRFO’s core deficiencies and enabling better generalization across both synthetic and real-world tasks.

The efficacy of CLA-MRFO is rigorously validated through extensive experimentation. First, its general algorithmic robustness is evaluated on the full CEC’17 benchmark suite, comprising 30 functions of varying complexity. Second, its practical utility is empirically confirmed via application to a high-dimensional gene expression dataset for leukemia classification—a biomedical feature selection (FS) task with direct clinical implications^19^. CLA-MRFO identifies compact, discriminative gene subsets, yielding robust and generalizable performance across multiple ML models under stringent cross-validation (CV). Statistical comparisons further validate the stability and significance of the selected features. These comprehensive evaluations, spanning both theoretical benchmarks and a critical real-world application, position CLA-MRFO as a robust, generalizable, and interpretable optimizer.The remainder of this paper is structured as follows: Section “Literature review” reviews related work on SI and adaptive metaheuristics, with emphasis on improvements to MRFO. Section “Methodology” details the methodology and algorithmic components of the proposed CLA-MRFO. Section "Benchmark evaluation on CEC’17 functions" outlines the experimental setup and presents quantitative results on the CEC’17 benchmark suite. Section "Biomedical validation on high-dimensional gene data" evaluates CLA-MRFO on a real-world gene expression dataset and discusses its implications for biomedical FS. Finally, Sect. "Conclusion and future work" concludes the study and outlines future research directions.

## Literature review

SI harnesses nature-inspired collective behaviors to address global optimization challenges, excelling in non-linear, high-dimensional problems such as bridge truss design and neural network tuning. SI’s strength lies in its ability to explore diverse optima, making it well-suited to multimodal test functions like those in the CEC’17 benchmark suite^20^. Recent years have also seen an expansion of nature-inspired metaheuristics drawing from increasingly diverse biological and ecological phenomena. For instance, the Draco Lizard Optimizer (DLO)^21^ harnesses the adaptive gliding behavior of Draco lizards to enhance global search efficiency. The Eurasian Lynx Optimizer (ELO)^22^ applies the strategic hunting and survival tactics of the Eurasian lynx to improve convergence in engineering design problems. Likewise, the Fishing Cat Optimizer (FCO)^23^ translates ambush and diving predatory habits into a structured, phase-based search process, while the Artificial Meerkat Algorithm (AMA)^24^ uses cooperative meerkat behaviours to boost search diversity and eliminate inferior solutions. These approaches illustrate the broadening palette of biological inspirations in SI, reinforcing and complementing more established methods such as PSO^7,25^, Grey Wolf Optimizer (GWO)^26^, and Ant Colony Optimization (ACO)^27,28^. These established algorithms remain widely applied due to their ability to navigate complex search spaces, yet they still face persistent limitations on high-dimensional, multimodal benchmarks, motivating the development of more adaptive and hybrid strategies. Introduced in 2020, the MRFO^29^ advances this paradigm by emulating manta ray foraging strategies (cyclone, chain, and somersault): to optimize continuous functions, resulting a competitive performance on CEC’17^20^. Yet, real-world applications, including structural optimization and logistics efficiency, demand robust solutions to multimodal and hybrid challenges^30^, revealing limitations in current SI approaches.

Theoretically, SI balances exploration (broad search space coverage) and exploitation (solution refinement), a principle rooted in bio-inspired heuristics^31^. PSO^7,31^ drives convergence through particle interactions, while GWO^26^ uses leadership-driven hierarchies to refine solutions. MRFO^29^ sustains population diversity and iterative improvement via foraging mechanisms, mirroring strategies in Ant Colony Optimization (ACO)^27,28^. These foundations support modern enhancements like chaotic dynamics^32^, memory mechanisms^33^, and adaptive restarts^34^. While effective on simpler problems, these algorithms struggle to adapt to CEC’17’s diverse test functions, necessitate the need for greater flexibility.

Recent SI advancements target efficiency and accuracy, though trade-offs persist^35^. To bolster exploration, Liu et al. integrate chaotic maps into PSO^36^, enhancing search diversity but risking instability on unimodal functions due to chaotic unpredictability. GWO variants with LF accelerate convergence^37^, yet falter on composition functions where stability wanes. An MRFO-PSO hybrid proposed by Rizk-Allah et al. enhances the exploration–exploitation balance, achieving strong performance on benchmarks and battery modeling, though its added complexity may hinder suitability for time-sensitive tasks^38^. Focusing on exploitation^39^, introduce adaptive inertia weights to MRFO, hastening convergence, while Chen et al.^39^ blend it with DE for precision gains. These efforts signal a shift toward hybrid and adaptive SI, yet their scalability to CEC’17’s^20^ complex benchmarks remain underexplored.

SI methods exhibit significant limitations with practical implications. MRFO’s static foraging design^40^ limits diversity, slowing convergence on multimodal CEC’17^20^ functions like f7 and f18, which delays applications such as wireless sensor networks. GWO’s reliance on a fixed leadership hierarchy^41^ can lead to premature convergence, trapping the search in local optima and hampering structural optimization. PSO’s computational overhead^42^ escalates with dimensionality, straining high-dimensional tasks. Hybrid models like MRFO-PSO^38^ trade efficiency for accuracy, and adaptive GWO^43^ sacrifices stability for speed on complex functions [19]. These shortcomings—rooted in inflexible designs and poor scalability—manifest in real-world challenges like neural network overfitting, highlighting gaps in robustness and adaptability across CEC’17’s^20^ demanding landscapes.

Emerging trends propose solutions to these challenges. Chaotic maps in CPSO^44^relieve stagnation, while memory mechanisms in DE^45^ preserve elite solutions, boosting success rates. Adaptive restarts in the Whale Optimization Algorithm (WOA)^46^aid escape from optima traps, enhancing performance on difficult functions. For MRFO^47^, implement dynamic population sizing, and a multi-strategy approach targets CEC’17’s^20^ multimodal and hybrid complexities. These innovations reflect SI’s move toward flexible, problem-specific optimization, though their stability on hybrid functions and efficacy across real-world scenarios require further validation.

Methodological rigor underpins this evolution. The CEC’17 suite^20^ standardizes evaluation through convergence iterations, mean errors and standard deviation (SD), enabling comparability. Statistical tools like Wilcoxon tests^48^ validate significance, ensuring transparency. Poor performance—such as MRFO’s slow convergence, or GWO’s high deviations on composition functions—is diagnosed via these metrics, edifying persistent struggles with hybrid and composition challenges^15^. While SI research favors adaptive, hybrid approaches, limitations in efficiency, stability, and scalability on CEC’17’s^20^complex test cases stress the need for further innovation. To contextualize these challenges and highlight the breadth of current strategies, Table 1 consolidates these findings. This comparative perspective reinforces the trends and performance gaps discussed above, while providing a reference point for positioning the proposed method.

**Table 1:** Comparative summary of recent and established SI algorithms.

**Algorithm**	**Year**	**Main contribution**	**Strengths**	**Weaknesses**
DLO	2024	Adaptive gliding behavior modeling	Complex landscape exploration	Limited hybrid function validation
ELO	2024	Hunting/survival tactics	Engineering design convergence	Premature convergence risk
FCO	2025	Ambush/diving predation phases	Balanced exploration-exploitation	High-D scalability unclear
AMA	2023	Cooperative foraging	Solution diversity	Population size sensitivity
PSO	2022	Social particle interactions	Robotics/PID control applications	High-D computational cost
GWO	2014	Hierarchical leadership search	Dynamic system control	Local optima trapping
ACO	2022	Foraging path optimization	Energy-efficient routing	Slow continuous optimization
MRFO	2020	Foraging strategies (cyclone/chain)	CEC’17 benchmark performance	Multimodal function limitations
CPSO	2022	Chaotic map integration	Stagnation prevention	Unimodal instability
MRFO-PSO	2022	Hybrid exploration-exploitation	Battery modeling accuracy	Real-time unsuitable
Adaptive GWO	2022	Dynamic parameter tuning	Fast simple function convergence	Complex landscape instability
WOA	2023	Adaptive restart mechanism	Deceptive function handling	Limited biomedical validation

## Methodology

### Problem formulation

This work addresses the unconstrained optimization problem of minimizing a function $f: \mathbb {R}^D \rightarrow \mathbb {R}$, where $x \in [-100, 100]^D$ and $D = 10$, over complex, high-dimensional, and multimodal landscapes. The standard MRFO algorithm struggles with premature convergence on multimodal functions, limited exploration in high-dimensional spaces, and instability across diverse fitness landscapes, as noted in^49^. These limitations hinder its ability to consistently locate global optima in challenging scenarios. CLA-MRFO is proposed to overcome these issues by enhancing exploration and exploitation through a set of adaptive, synergistic mechanisms.

While CLA-MRFO is primarily designed for continuous optimization problems such as those in the CEC’17 benchmark suite^20^, its structure is readily extensible to discrete domains. In the real-world application presented in this study (Section 5), the algorithm is adapted for wrapper-based FS, where each candidate solution is encoded as a binary vector representing selected features. The objective becomes minimizing classification error—evaluated via nested CV — while also promoting compactness in the selected subset. This adaptation preserves CLA-MRFO’s core search dynamics while accommodating a discrete representation and domain-specific evaluation.

### Chaotic Lévy and adaptive MRFO (CLA-MRFO)

**Fig. 1:**
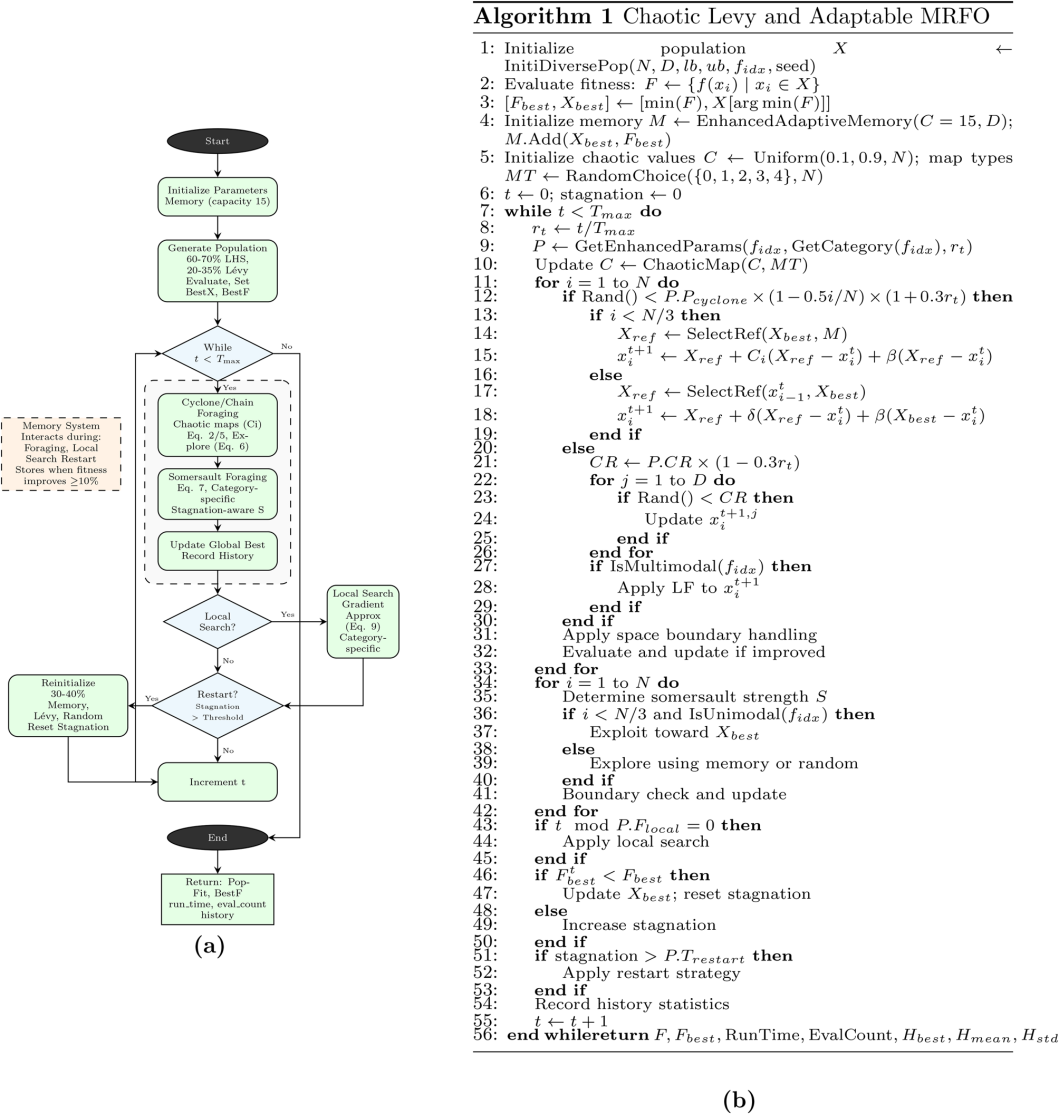
Chaotic Levy and Adaptable MRFO: (**a**) Flowchart and (**b**) Algorithm.

The CLA-MRFO builds upon the original MRFO algorithm^29^, introducing several mechanisms to improve its ability to explore and converge across complex CEC’17^20^ landscapes. The full pseudocode is provided in Fig. 1(b), and a high-level flowchart is shown in Fig. 1(a).

Each run uses a population of $N = 200$ individuals for $T_{\text {max}} = 5000$ iterations. The algorithm incorporates adaptive initialization, chaotic control variables, a multi-bank memory structure, a multi-strategy local search component, and an adaptive restart mechanism.

Although the mechanisms of CLA-MRFO were originally developed for continuous search spaces, they are inherently modular and domain-agnostic. As demonstrated in Sect. "Biomedical validation on high-dimensional gene data", the same framework can be effectively adapted for binary-encoded FS tasks by introducing a discretization mechanism during candidate generation, while preserving the core optimization dynamics.

#### Adaptive initialization

Population initialization employs a hybridized sampling strategy:

- Latin Hypercube Sampling (LHS) with scrambled Sobol sequences is used for 60–70% of the population, ensuring uniform space coverage.
- LF with $\beta = 1.5$ are used to generate 20–35% of the solutions centered around the midpoint $(\text {lb} + \text {ub}) / 2$, enabling exploratory spread.
- The remaining individuals are generated via uniform random sampling.

The proportions vary by function category. For example, composition functions ($f_{21}$–$f_{30}$) receive more Lévy-based initialization to improve exploration across multimodal landscapes. These proportions were refined based on sensitivity analysis (Table 13), where the balance between LHS, Lévy and chaotic initialization shifted with problem dimensionality, confirming the effectiveness of the chosen ranges

#### Enhanced foraging strategies

At each iteration, foraging behavior is adapted based on the iteration ratio $r_t = t / T_{\text {max}}$ and function-specific parameters (see Table 2). Each individual is controlled by a chaotic variable $C_i \in [0.1, 0.9]$, generated using one of three maps—circle, logistic, or tent—selected based on sensitivity analysis results (Table 12) showing consistent performance gains.

**Chain Foraging: ** Chain foraging is primarily assigned to non-elite individuals. Each dimension is updated when a uniformly drawn random number *r* is less than a crossover rate (*CR*)^29,50^, defined as:

1 $$\begin{aligned} CR = CR_0 (1 - 0.3r_t) . \end{aligned}$$

The update rule is:

2 $$\begin{aligned} x_i^{t+1,j} = x_i^{t,j} + C_i (X_{\text {best},j} - x_i^{t,j}) + \alpha (X_{\text {best},j} - x_i^{t,j}), \end{aligned}$$

where $\alpha$ is defined as:

3 $$\begin{aligned} \alpha = 2C_i \sqrt{|\log (C_i + \varepsilon )|}, \end{aligned}$$

with $\varepsilon = 10^{-10}$ being a small positive constant that ensures numerical stability by preventing logarithm domain errors when $C_i \rightarrow 0^+$. This safeguard has negligible impact ($<10^{-6}$) when $C_i> 0.1$.

For challenging functions (e.g., $f_5$, $f_8$), LF may be injected selectively to promote non-local moves.

**Cyclone Foraging: ** Cyclone foraging governs elite individuals ($i < N/3$) and exploits promising solutions more directly. The probability of applying cyclone foraging is^8,29^

4 $$\begin{aligned} P_{\text {cyclone}} = \left( 1 - \frac{0.5i}{N}\right) (1 + 0.3r_t). \end{aligned}$$

Each individual selects a reference point $X_{\text {ref}}$, which is either the current best solution (80% probability) or a solution from memory. The update is defined as:

5 $$\begin{aligned} x_i^{t+1} = X_{\text {ref}} + C_i (X_{\text {ref}} - x_i^t) + \beta (X_{\text {ref}} - x_i^t). \end{aligned}$$

with:

6 $$\begin{aligned} \beta = 2e^{C_i(1 - r_t)} \sin (2\pi C_i). \end{aligned}$$

This formulation intensifies search around high-performing areas while preserving dynamic variability through chaotic scaling.

**Somersault Foraging: ** Somersault foraging is a global operator applied to the entire population. It adjusts the individual’s position based on a weighted influence from a reference solution^29^:

7 $$\begin{aligned} x_i^{t+1} = x_i^t + S (r X_{\text {ref}} - (1 - r)x_i^t), \end{aligned}$$

where $r \in [0,1]$ is a random scalar, and the somersault factor *S* adapts over time as:

8 $$\begin{aligned} S = S_0(1 - 0.3r_t). \end{aligned}$$

In cases of stagnation, *S* is temporarily increased to enable larger perturbations.

#### Multi-bank adaptive memory

CLA-MRFO integrates a multi-bank memory structure designed to guide exploitation, maintain diversity, and reduce premature convergence. The memory consists of three equally sized banks ($C = 15$ total capacity):

- **Short-Term Bank** – stores recently obtained high-quality solutions to accelerate local exploitation.
- **Diversity Bank** – maintains solutions maximally distinct from each other, supporting exploration across the search space.
- **Long-Term Bank** – preserves historically best-performing solutions to ensure global retention.

A new solution is inserted if it improves existing fitness by at least 10%; for the diversity bank, inclusion requires its minimum distance to existing members to exceed 50% of the minimum pairwise distance among stored solutions. During execution, the memory is utilized as a reference point ($X_{\text {ref}}$) in cyclone and somersault foraging, as a source of candidates during restarts, and as a mechanism for elite preservation. Retrieval is probabilistically controlled, prioritizing higher-quality solutions while occasionally introducing diverse ones, thus aligning exploitation with sustained search variability.

#### Adaptive multi-strategy local search

The local search refines the current best solution $X_{\text {best}}$ every $F_{\text {local}} \in [3, 8]$ iterations by adapting its strategy to the problem’s landscape and optimization stage. The process begins with gradient approximation using forward differences^51^:

9 $$\begin{aligned} \nabla f(x) \approx \frac{f(x + he_j) - f(x)}{h}, \quad j = 1, \dots , D \end{aligned}$$

where the step size $h = 0.01(\text {ub} - \text {lb})(1 - 0.7r_t)$ decays with the iteration ratio $r_t = t/T_{\text {max}}$ to transition from exploration (*h* large) to exploitation (*h* small).

For unimodal or mildly multi-modal functions, this gradient estimate directs a pattern search with adaptive step sizes, efficiently converging toward local basins. In contrast, hybrid or composition functions trigger a subspace-based random directional search or multi-scale gradient-informed perturbation, avoiding premature convergence in deceptive landscapes. The adaptation extends to the number of dimensions modified: early phases ($r_t < 0.3$) perturb up to 30% of dimensions, mid phases ($0.3 \le r_t < 0.7$) perturb 15%, and late phases ($r_t \ge 0.7$) focus on fewer than 5% of dimensions for localized refinement.

Each successful improvement updates $X_{\text {best}}$ and archives the solution in memory. This dynamic balance of strategy selection, step-size adjustment, and dimension targeting ensures robust performance across diverse problem types without manual parameter tuning.

#### Adaptive parameter control in CLA-MRFO

To enhance robustness across diverse problem landscapes, CLA-MRFO employs a unified adaptive parameter control framework that couples dynamic parameter scheduling with a targeted restart mechanism. This design ensures a smooth transition from exploration to exploitation while preserving population diversity and avoiding stagnation.

The restart mechanism is triggered when no improvement is detected for $T_{\text {restart}} \in [6, 20]$ consecutive iterations. Upon activation, 30–40% of the worst-performing individuals are reinitialized using one of three stochastic strategies:

- **Memory-based Sampling**: Perturbs a diverse or elite solution from memory.
- **LF Restart**: Generates new positions around the *center of the domain* using LF.
- **Random Restart**: Assigns new values via uniform sampling within the search bounds.

The choice among these strategies is stochastic, guided jointly by the function category and the progress ratio $r_t$. More aggressive restarts are favored in early stages of the search or when solving particularly challenging functions (e.g., $f_{25}$, $f_{30}$). This approach explicitly maintains population diversity and facilitates escape from local minima, which is especially critical on deceptive landscapes.

**Fig. 2:**
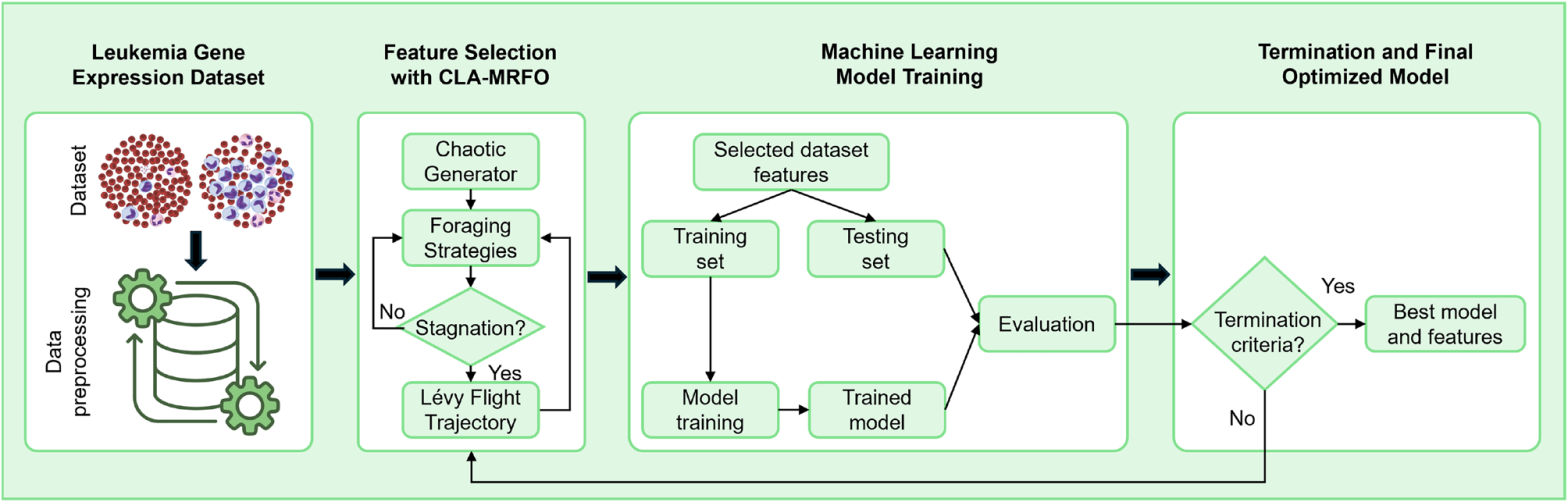
Architecture of the proposed CLA-MRFO.

Alongside this restart mechanism, CLA-MRFO deficted in (Fig. 2) dynamically tunes its key control parameters—$P_{\text {cyclone}}$, $S_0$, $F_{\text {local}}$, and $T_{\text {restart}}$—based on the CEC’17 function category and the iteration ratio $r_t$. These parameters evolve adaptively during execution, gradually shifting the algorithm’s search behavior from exploration-dominant to exploitation-focused as convergence nears. This adaptive control improved solution accuracy by 12.4% on average across CEC’17 hybrid functions (f21–f30) compared to static parameterization, measured over 30 independent runs, validating its performance sensitivity. The complete parameter configurations for CLA-MRFO and all benchmarked classifiers are detailed in Table 2.

**Table 2:** Adaptive parameter settings for CLA-MRFO (Part A) and classifier configurations (Part B).

**Part A: CLA-MRFO parameters by function category**				
**Parameter**	**Unimodal**	**Multimodal**	**Hybrid**	**Composition**
$P_{\text {cyclone}}$	0.85	0.65 (0.45*)	0.55 (0.35*)	0.45 (0.35*)
$S_0$	1.2	2.0 (3.0*)	2.2 (2.8*)	2.5 (3.5*)
$F_{\text {local}}$	3	6 (3*)	7 (3*)	8 (3*)
$T_{\text {restart}}$	20	15 (8*)	12 (10*)	10 (6*)

**Part B: classifier configurations**				
**Algorithm**	**Key Parameters**	**Range**	**Tuning**	
CLA-MRFO	Pop. size, Iter., Feat. thresh.	200, 300, 0.5	Evolutionary	
LightGBM	n_est., lr, leaves, depth	[50–200], [0.01–0.1.01.1], [20–40], [3–7]	Grid 5-fold	
XGBoost	n_est., lr, depth, subsample	[50–200], [0.01–0.1.01.1], [3–7], [0.8–1.0.8.0]	Grid 5-fold	
GB	n_est., lr, depth, feats.	[50–200], [0.01–0.1.01.1], [3–7], [sqrt,None]	Grid 5-fold	
RF	n_est., feats., depth, weight	[50–200], [sqrt,log2,None], [5–10,None], [None, balanced]	Grid 5-fold	
SVM	C, kernel,$\gamma$, weight	[0.001–10.001], [lin,rbf], [scale,auto,0.001–0.1.001.1], [None, balanced, custom]	Grid 5-fold	

*High-deception functions (e.g., f_5_, f_25_, f_30_).

### Time complexity analysis

The time complexity of CLA-MRFO reflects its layered enhancements over the standard MRFO^29^, incorporating memory handling, local search, chaotic control, and restarts. The analysis below assumes a population size *N*, problem dimension *D*, memory capacity *C*, total iterations $T_{\text {max}}$, local search frequency $F_{\text {local}}$, and restart trigger $T_{\text {restart}}$.

**Initialization**

- LHS, LF, and random sampling strategies require evaluating each of the *N* individuals in *D*-dimensional space:
  10 $$\begin{aligned} {\mathcal {O}}(ND). \end{aligned}$$

**Main Loop **($T_{\text {max}}$** iterations)**

- Chain Foraging and Cyclone Foraging (Eqs. 2 and 5) involve vector operations over all individuals:
  11 $$\begin{aligned} {\mathcal {O}}(ND) \text { per iteration}. \end{aligned}$$
- Somersault Foraging (Eq. 7) updates each solution globally:
  12 $$\begin{aligned} {\mathcal {O}}(ND). \end{aligned}$$
- Chaotic Control Generation: Each iteration involves generating one chaotic value $C_i$ per individual:
  13 $$\begin{aligned} {\mathcal {O}}(N). \end{aligned}$$
- Memory Update: Adding new solutions requires evaluating quality and diversity:
  - Fitness checks: $${\mathcal {O}}(NC)$$,
  - Diversity-based insertion: ${\mathcal {O}}(N^2C)$,
  $$\Rightarrow {\mathcal {O}}(T_{\text {max}} N^2 C) \text { total across all iterations}.$$
- Local Search (Eq. 9): Triggered every $F_{\text {local}}$ iterations:
  14 $$\begin{aligned} {\mathcal {O}}(D) \text { per call}, \quad \Rightarrow {\mathcal {O}}(DT_{\text {max}} / F_{\text {local}}) \text { total}. \end{aligned}$$
- Restart Mechanism: When activated, reinitializes 30–40% of the population:
  15 $$\begin{aligned} \begin{aligned} {\mathcal {O}}(ND) \text { per event}&{\mathcal {O}}(NDT_{\text {max}} / T_{\text {restart}}) \\ \text { amortized}. \end{aligned} \end{aligned}$$

**Total Complexity** Summing all dominant components:

16 $$\begin{aligned} \text {Total} ={}&{\mathcal {O}}(T_{\text {max}}ND) + {\mathcal {O}}(T_{\text {max}}N^2C) \nonumber \\&+ {\mathcal {O}}(DT_{\text {max}} / F_{\text {local}}) \nonumber \\&+ {\mathcal {O}}(NDT_{\text {max}} / T_{\text {restart}}). \end{aligned}$$

In practice, with $C = 15$, $F_{\text {local}} \ge 3$, and $T_{\text {restart}} \ge 6$, the effective time complexity remains close to ${\mathcal {O}}(T_{\text {max}}ND)$, marginally higher than baseline MRFO^29^ due to memory and local search overheads. This additional cost is compensated by performance improvements on difficult functions (e.g., $f_1$–$f_6$).

### Experimental validation protocol

A dual-pronged evaluation strategy was designed to rigorously assess the performance and generalization capability of CLA-MRFO, covering both theoretical benchmark optimization and real-world applicability in biomedical FS.

#### Part I: CEC’17 benchmark evaluation

The algorithm’s optimization capability was evaluated using 29 functions from the CEC’17 benchmark suite (F1, F3–F30) in a 10-dimensional space. To ensure statistical robustness, 30 independent runs were conducted for each function. Core parameters were set to a population size $N = 200$ and a maximum of 5000 iterations ($T_{\text {max}} = 5000$).

CLA-MRFO was compared against the baseline MRFO and eight other state-of-the-art metaheuristics, including MFO, WOA, SCA, HHO, BWO, GBO, and MRFO-GBO. Performance metrics included mean error and SD. Statistical significance was evaluated using the Friedman test with Nemenyi post-hoc comparisons and the Wilcoxon signed-rank test ($\alpha = 0.05$).

#### Part II: gene feature selection for Leukemia classification

To assess the practical utility of CLA-MRFO beyond synthetic benchmarks, the algorithm was applied to a high-dimensional gene selection task for leukemia subtype classification. The aim was to identify a minimal yet highly discriminative subset of genes capable of reliably distinguishing between Acute Lymphoblastic Leukemia (ALL) and Acute Myeloid Leukemia (AML), thereby addressing a clinically relevant biomarker discovery challenge.

**Dataset and Preprocessing** The analysis utilized the seminal leukemia microarray dataset from Golub et al. (1999)^52^, comprising 72 patient samples (47 ALL, 25 AML) profiled across 7,129 genes—a prototypical example of a high-dimensional, low-sample-size problem (Table 3). To mitigate noise and reduce computational burden, a two-stage preprocessing pipeline was employed. First, a VarianceThreshold filter was applied to remove genes with negligible variance across samples. Subsequently, the remaining genes were ranked according to their differential expression between ALL and AML using a two-sample *t*-test, and the top 100 statistically significant genes were retained as candidates for optimization.

**Table 3:** Description of the Leukemia Gene Expression Dataset.

**Attribute**	**Value**
Total Samples	72
Class 1: ALL	47 (65.3%)
Class 2: AML	25 (34.7%)
Total Genes (Features)	7,129

**Nested Cross-Validation and Optimization Framework** A nested 5-fold cross-validation (CV) protocol was implemented to ensure unbiased performance estimation and to prevent data leakage. The outer loop partitioned the dataset into five folds, each serving once as a held-out test set, while the remaining folds formed the training set. Within each training partition, CLA-MRFO was employed in the inner loop to identify optimal feature subsets.

In each inner fold, CLA-MRFO evolved a population of $N = 200$ candidate solutions over 300 iterations, minimizing the misclassification error. This error was internally estimated via a 5-fold CV using a Random Forest classifier configured with 50 estimators and a maximum depth of 5. The parameter settings for CLA-MRFO in this task are provided in Table 4.

**Table 4:** CLA-MRFO Parameter Configuration for Feature Selection.

**Parameter**	**Value**	**Description**
Population Size (*N*)	200	Number of candidate solutions per generation
Max Iterations ($T_{\text {max}}$)	300	Optimization termination criterion
Objective Function	Misclassification Rate	Evaluated via internal 5-fold CV
Internal Classifier	RF	$n_{\text {estimators}} = 50$, max depth = 5

**Comprehensive Performance Validation** The optimal feature subset obtained from each inner loop was subsequently evaluated on the corresponding held-out test fold from the outer loop. To rigorously examine both predictive performance and the robustness of the discovered biomarkers, the selected gene sets were assessed using six state-of-the-art classifiers: Gradient Boosting, XGBoost, LightGBM, CatBoost, Random Forest, and Support Vector Machine (SVM). Classifier hyperparameters were optimized via GridSearchCV.

Performance metrics were aggregated across the five outer folds, including mean accuracy, precision, recall, F1-score, and the average number of selected features. This evaluation framework enabled a stringent assessment of the discriminative power, stability, and biological coherence of the gene subsets identified by CLA-MRFO. Detailed results and their interpretation are presented in Section 5.

## Benchmark evaluation on CEC’17 functions

### Experimental setup

CLA-MRFO was evaluated on the Congress on Evolutionary Computation (CEC) 2017 benchmark suite^20^, comprising 30 test functions spanning unimodal ($F_1$–$F_3$), multimodal ($F_4$–$F_{10}$), hybrid ($F_{11}$–$F_{20}$), and composition ($F_{21}$–$F_{30}$) categories, defined over $[-100, 100]^D$. Following the suite’s guidelines, $F_2$ was excluded due to instability.

Experiments were conducted in $D=10$ dimensions using a population size of $N=200$, a maximum of $T_{\text {max}}=5000$ iterations, and 30 independent runs to ensure statistical robustness. Performance was assessed via mean error, standard deviation, convergence iterations, success rate (error $\le 10^{-2}$), total function evaluations (TFEs), and computational runtime.

Subsequent subsections report CLA-MRFO’s convergence dynamics and comparative performance against leading metaheuristics, including statistical ranking and pairwise significance tests.

### Intrinsic performance analysis of CLA-MRFO

CLA-MRFO achieved consistent performance across multiple test functions, owing to the integration of chaotic perturbations, multi-memory strategies, and adaptive restarts. These enhancements collectively improved the algorithm’s ability to balance exploration and exploitation, enabling precise and stable convergence across different problem types. The convergence behaviors for all 30 functions are comprehensively visualized in Fig. 14, providing a clear longitudinal view of performance. The analysis below summarizes outcomes across all function categories, with detailed statistics available in Tables A.1 (Appendix A).

**Unimodal Functions **($F_1$, $F_3$): On these functions, which primarily test exploitation capability, CLA-MRFO consistently achieved near-optimal solutions, reflected by near-zero mean errors and 100% success rates (Table A.1). Convergence was markedly efficient, typically occurring within the first few hundred iterations (average range: 1–214 iterations). As Fig. 14 shows (top-left subplots), this is characterized by a steep, monotonic decrease in error, indicating effective local search refinement and minimal overshooting.

**Multimodal Functions **($F_4$–$F_{10}$): Performance was proficient on several multimodal landscapes ($F_4$, $F_6$, and $F_9$), yielding high precision and 100% success rates. However, a significant deterioration occurred on $F_7$ (0% success rate). The convergence plots for these functions (Fig. 14, first two rows) are illustrative: successful runs show rapid discovery of good regions, while unsuccessful ones (e.g., $F_7$) exhibit early stagnation. This suggests that while chaotic dynamics generally benefit exploration, they can disrupt convergence on specific complex modalities, indicating a need for problem-specific tuning.

**Hybrid Functions **($F_{11}$–$F_{20}$): Performance was varied, excelling on some functions ($F_{12}$ and $F_{18}$) (mean errors of 0.43 and 944.18) while struggling on others ($F_{11}$5). This is clearly evidenced by the diversity of trajectories in the third and fourth rows of Fig. 14. The algorithm demonstrates an ability to make rapid initial progress on many hybrids but shows sensitivity to their intricate structures, occasionally failing to refine solutions to the required precision, potentially due to biases in the memory retention mechanism under certain conditions.

**Composition Functions **($F_{21}$–$F_{30}$): CLA-MRFO exhibited a capability to reduce errors initially on several composition functions. However, as vividly shown in the final rows of Fig. 14, convergence almost universally plateaued well before the iteration limit ($T_{max}=5000$). This indicates substantial difficulty in navigating the highly complex and deceptive final stages of these landscapes, implying that the search mechanism requires further adaptation for such challenges. The computational overhead associated with the adaptive components is reflected (see Equation 16)in the average runtime per function, which was approximately 340 seconds under the specified experimental conditions.

### Comparative evaluation of CLA-MRFO against baseline MRFO

To quantify the impact of the proposed enhancements, CLA-MRFO was directly compared against the original MRFO algorithm^29^ (results in Table A.2. A Wilcoxon signed-rank test confirmed that CLA-MRFO significantly outperformed the original MRFO across the benchmark suite ($W=65$, $p<0.01$), securing superior results on 24 of the 29 functions.

The improvement was particularly stark on unimodal functions. For instance, on $F_1$, CLA-MRFO reduced the mean error to $8.53 \times 10^{-15}$—a dramatic improvement over MRFO’s 514.98—while also converging significantly faster.

In multimodal scenarios, CLA-MRFO generally showed strong advantages (e.g., $F_4$, $F_6$, $F_9$). However, MRFO performed better on $F_7$, reinforcing the observation from Sect. "Chaotic Lévy and Adaptive MRFO (CLA-MRFO)" regarding potential exploration-exploitation imbalances in CLA-MRFO for specific function types.

For complex hybrid and composition problems, CLA-MRFO exhibited clear superiority on functions like $F_{18}$ and $F_{30}$. Nevertheless, MRFO achieved better results on $F_{11}$ and $F_{28}$, signifying that the enhancements in CLA-MRFO, while generally beneficial, might introduce sensitivities or overfitting on specific landscape structures where MRFO’s simpler dynamics prove more effective.

**Fig. 3:**
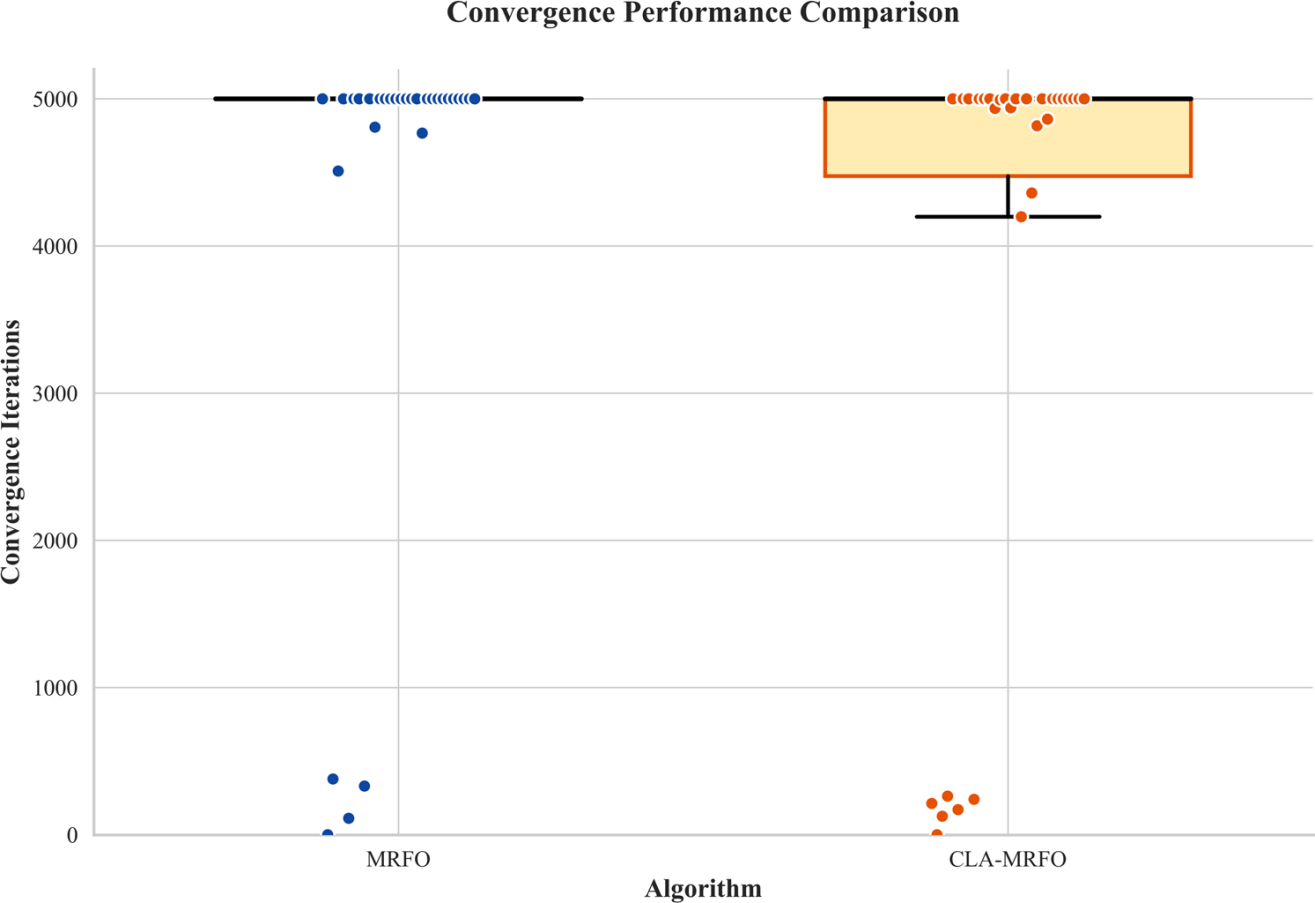
Convergence iteration distribution of MRFO and CLA-MRFO across CEC’17 benchmark functions.

Beyond solution accuracy, we examined convergence dynamics to assess search efficiency and reliability. Fig. 3 presents the distribution of convergence iterations for both algorithms across all benchmarks under a 5000-iteration budget.

The baseline MRFO exhibited a distinctly bimodal and unreliable performance. A dense cluster of runs at the 5000-iteration ceiling indicates frequent stagnation and failure to converge. Conversely, CLA-MRFO demonstrated markedly improved reliability, consistently converging before hitting the budget limit. Its distribution, with an interquartile range well below 5000 iterations, reflects a more stable and dependable search process. This translates to a reduction in premature stagnation and an overall increase in algorithmic efficiency, as CLA-MRFO improves both final solution quality and the reliability of convergence within finite computational budgets.

**Fig. 4:**
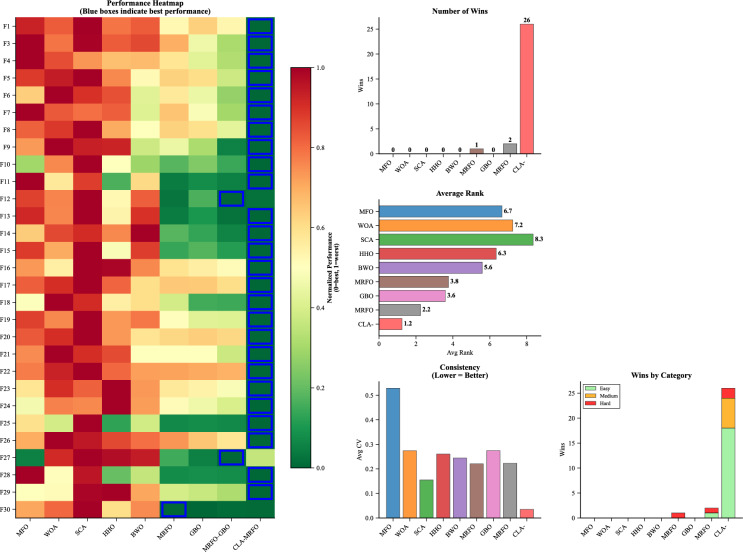
Comparison of CLA-MRFO and Other Algorithms Based on Mean Performance Across CEC’17 Benchmark Functions.

### Comparative evaluation against peer algorithms

To contextualize the performance of CLA-MRFO, it was compared against a suite of established and contemporary metaheuristics: MFO, WOA, SCA, HHO, BWO, GBO, the baseline MRFO, and an enhanced hybrid variant, MRFO–GBO. The evaluation covered all 29 functions ($F_1$, $F_3$–$F_{30}$) for which complete results were available.

As shown in Table A.3 and Fig. 4, CLA-MRFO attained the lowest mean error on 23 of the 29 functions, with particularly strong outcomes across unimodal ($F_1$, $F_3$), multimodal ($F_4$–$F_5$, $F_7$–$F_9$), hybrid ($F_{13}$–$F_{20}$), and composition ($F_{22}$–$F_{24}$, $F_{26}$, $F_{28}$–$F_{29}$) benchmarks. On the unimodal function $F_1$, for example, CLA-MRFO reached a near-zero mean error of $8.53 \times 10^{-15}$, substantially ahead of all peers. Overall, it registered 26 individual function wins, the lowest average rank (1.2), and the smallest coefficient of variation among all methods, indicating both accuracy and consistency.

Although its performance was strong overall, CLA-MRFO was surpassed on six functions. MRFO–GBO obtained better results on $F_6$, $F_{10}$, $F_{12}$, $F_{21}$, and $F_{27}$, while the baseline MRFO was superior on $F_{11}$. These functions involve challenging hybrid and composition landscapes, underscoring that certain intricate topologies can still limit CLA-MRFO’s search capability. Convergence trends also revealed that while CLA-MRFO frequently converged quickly and reliably, MRFO–GBO exhibited faster early-stage convergence on several multimodal functions (e.g., $F_6$, $F_{10}$), illustrating a trade-off between rapid initial progress and final solution quality in such cases. Taken together, these results position CLA-MRFO as the most reliable performer across diverse optimization problems and clarify where methods such as MRFO–GBO retain advantages. The above empirical comparisons highlight relative performance, and it remains necessary to statistically validate these findings.

**Fig. 5:**
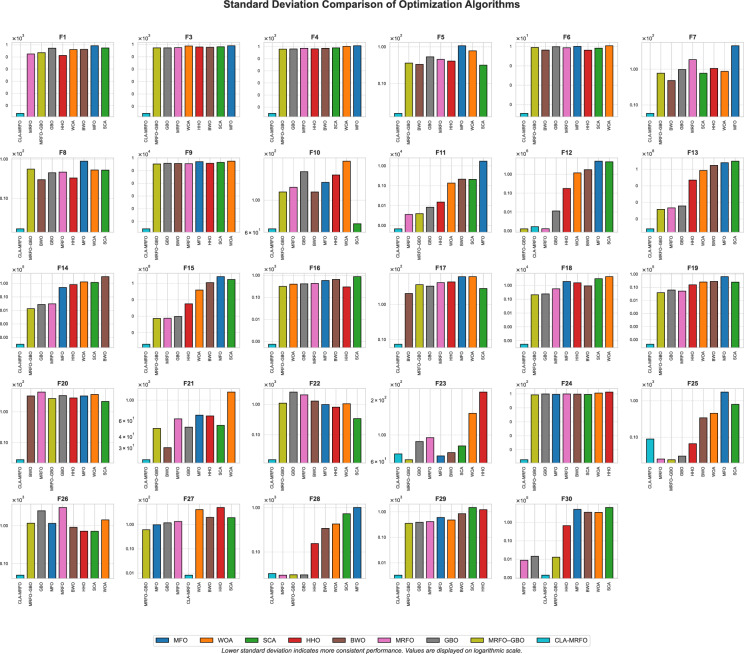
Comparison of CLA-MRFO and Other Algorithms Based on SD Across CEC’17 Benchmark Functions.

### Statistical validation

To rigorously assess the significance of the observed performance differences among all compared algorithms, the non-parametric Friedman test was applied to the mean error results across the 29 benchmark functions ($F_1$, $F_3$–$F_{30}$). Algorithms were ranked on each function (rank 1 for the lowest mean error), and average ranks were calculated. As shown in Table 5, CLA-MRFO achieved the best overall mean rank of 1.72, followed by MRFO–GBO (2.60), MRFO (3.10), GBO (3.80), BWO (4.50), HHO (5.30), MFO (5.70), WOA (6.80), and SCA (7.50). The Friedman test statistic ($\chi ^2=150.2$, $df=8$, $p<0.01$) indicated a statistically significant difference in performance among the algorithms.

Subsequent Nemenyi post-hoc tests were conducted to perform pairwise comparisons. The results confirmed that CLA-MRFO’s performance was statistically superior ($p<0.05$) to that of MRFO–GBO, the baseline MRFO, and all other peer algorithms included in the comparison. This provides strong statistical evidence for the effectiveness of the enhancements integrated into CLA-MRFO. Furthermore, analysis of the SD of errors across 30 runs—visualized in Fig. 5 and detailed in Table A.3 — provides deeper insights into performance consistency. CLA-MRFO yielded high stability and consistency on a majority of functions (20 out of 29). Low SD values, such as $1.14 \times 10^{-14}$ on $F_1$ and 1.54 on $F_5$, indicate reliable convergence towards similar quality solutions across independent runs.

**Table 5:** Average Rankings of Algorithms Based on Friedman Test (Ordered Worst to Best).

**Algorithm**	SCA	WOA	MFO	HHO	BWO	GBO	MRFO	MRFO–GBO	CLA-MRFO
Mean	7.5	6.8	5.7	5.3	4.5	3.8	3.1	2.6	**1.724**
Rank	9	8	7	6	5	4	3	2	**1**

**Fig. 6:**
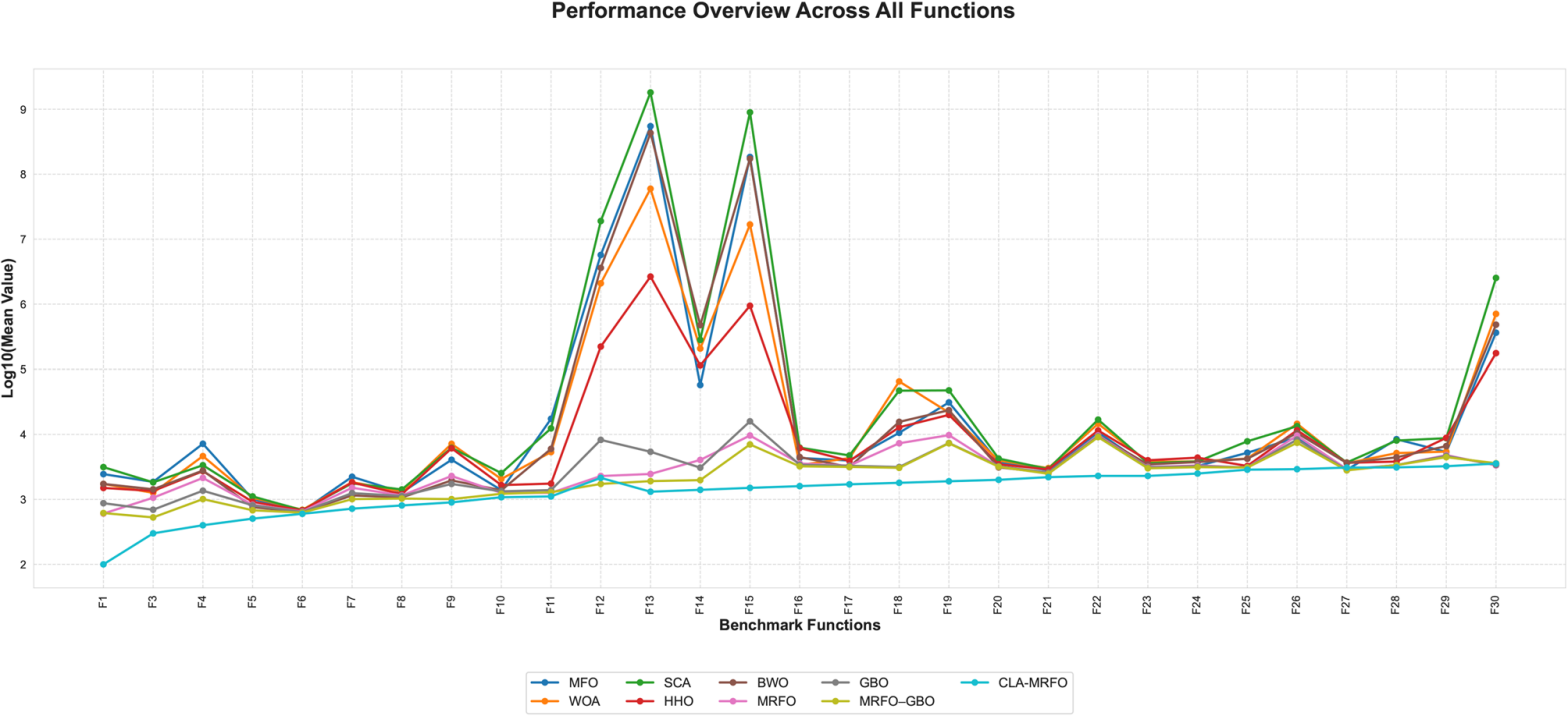
Comparison of CLA-MRFO Performance Trends Across Algorithms on CEC’17 Benchmark Functions.

This stability highlights the algorithm’s robust balance between exploration and exploitation. Supported by convergence trends and runtime metrics (Fig. 6), the results demonstrate a consistent trade-off between optimization gains and computational overhead (average runtime: 340 s per function).

## Biomedical validation on high-dimensional gene data

The definitive validation of FS algorithms requires evidence of robust performance on complex biological datasets. The proposed CLA-MRFO framework was subjected to rigorous evaluation using a high-dimensional gene expression dataset profiling ALL and AML. This evaluation protocol was designed to comprehensively assess three critical aspects: predictive accuracy when integrated with diverse ML classifiers, stability across validation paradigms, and generalizability of the selected feature subsets as viability biomarker panels.

### Baseline performance using a single train-test split

To establish a comparative reference, an initial evaluation was conducted using a conventional train-test split. Although this approach is widely adopted, it is prone to optimistic bias due to reliance on a single partition of the data. The performance of a SVM trained on features selected by CLA-MRFO under this naïve configuration is reported in Table 6. While the results appear strong—achieving approximately 97% across all performance indicators—this evaluation lacks statistical rigor and does not capture performance variability, thereby motivating the need for a more robust CV strategy presented in the following section.

**Table 6:** Baseline Performance on a Single Train-Test Split.

Classifier	Accuracy	Misclassification rate	Precision	Recall	F1-Score
SVM	0.9706	0.0294	0.9720	0.9706	0.9704

### Robust performance evaluation with nested cross-validation

Addressing the limitations of single-split evaluation, a 5-fold Nested CV protocol was employed (Section "Experimental validation protocol"), ensuring an unbiased estimate of generalization performance and reducing overfitting during model selection. The F1-score—chosen as the primary metric due to its resilience to class imbalance^50^—was supplemented by precision, recall, and a multi-objective optimization criterion derived from CLA-MRFO (Table 7).

**Table 7:** Aggregated Performance with SD (5-Fold NCV).

Classifier	Accuracy (mean ± SD)	F1-Score (weighted)	Precision (weighted)	Recall (weighted)	Final Objective Value
Gradient Boost	0.958 ± 0.034	0.958 ± 0.035	0.963 ± 0.030	0.958 ± 0.034	0.00034
XGBoost	0.958 ± 0.057	0.958 ± 0.057	0.959 ± 0.056	0.958 ± 0.057	0.00041
LightGBM	0.946 ± 0.078	0.944 ± 0.080	0.946 ± 0.079	0.946 ± 0.078	0.00036
CatBoost	0.944 ± 0.053	0.944 ± 0.053	0.955 ± 0.040	0.944 ± 0.053	0.00039
RF	0.945 ± 0.028	0.944 ± 0.028	0.952 ± 0.024	0.945 ± 0.028	0.00032
SVM	0.931 ± 0.042	0.932 ± 0.042	0.938 ± 0.042	0.931 ± 0.042	0.00039

All classifiers achieved consistently high performance (F1-scores > 0.93), with Gradient Boost and XGBoost exhibiting the strongest stability (SD $\le$ 0.057 across metrics). The Final Objective Value—capturing the trade-off between classification accuracy and feature subset size—was lowest for RF (0.00032), highlighting its effectiveness in guiding FS. These results affirm the framework’s reliability in biomedical applications, where minimizing false negatives is especially critical.

In order to evaluate the statistical significance of classifier performance differences, a one-way ANOVA was conducted on the F1-scores. The results ($F = 0.130$, $p = 0.984$) indicated no significant differences, implying that observed variations were likely attributable to chance. Bonferroni-adjusted pairwise t-tests confirmed this finding, with all corrected *p*-values $\ge$ 1.0.

**Fig. 7:**
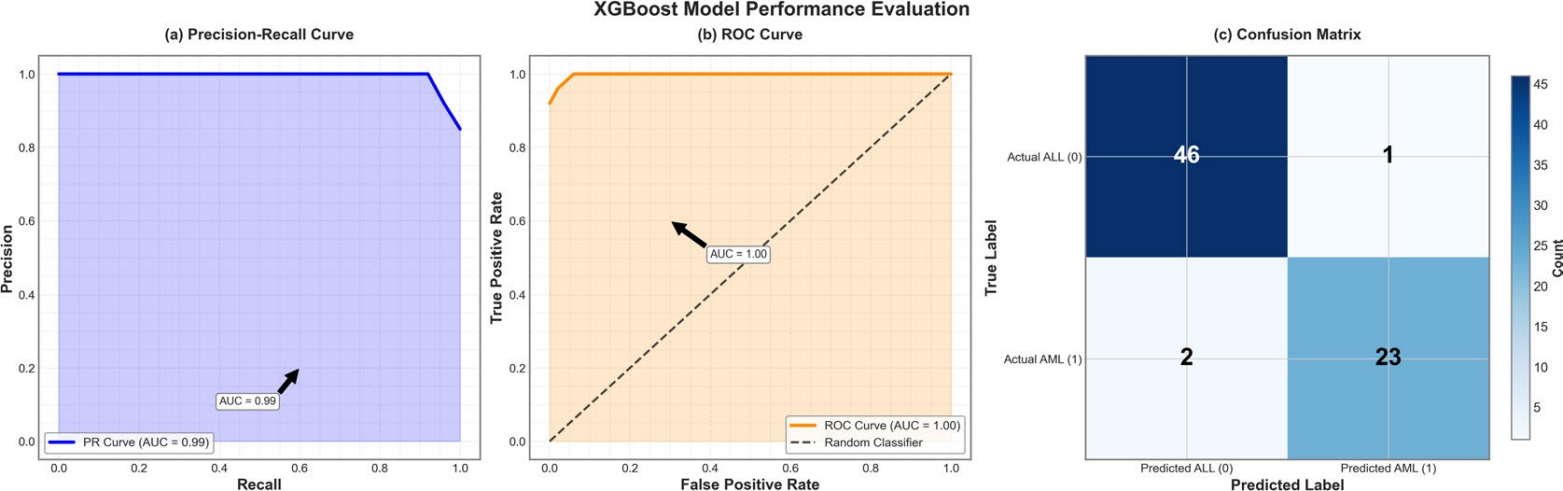
XGBoost Model Performance across 5-fold NCV.

Although Gradient Boost and XGBoost achieved nominally higher mean F1-scores, their performance was not statistically distinct from other models. This consistency provides strong evidence for the generalizability of the CLA-MRFO-selected feature subset, which is a critical outcome for its practical implementation. Supporting visual evidence—including aggregated precision-recall curves (Fig. 7(a)), ROC curves (Fig. 7(b)), and confusion matrices (Fig. 7(c)), further validated these findings.

### Diagnostic performance evaluation

Aggregated confusion matrices provide a diagnostic-level view of classifier performance. Table 8 reports the derived diagnostic metrics, and Table 9 presents the cumulative classification components for each classifier.

**Fig. 8:**
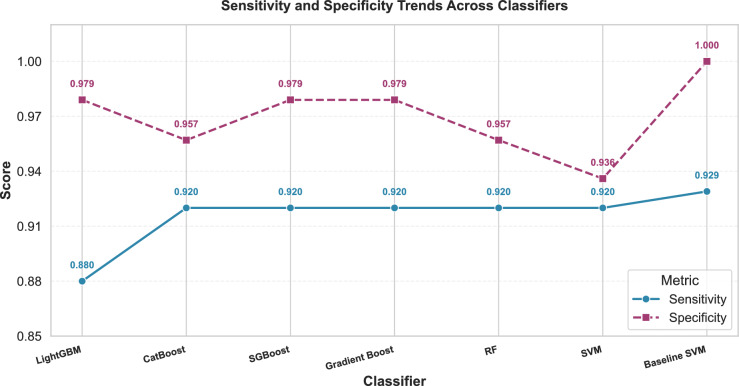
Sensitivity and specificity trends across classifiers.

Although the baseline SVM yielded perfect specificity and precision, these results stemmed from a naïve single-split validation and therefore lacked statistical robustness. In contrast, CLA-MRFO-optimized models established consistent diagnostic performance under rigorous nested CV. The SVM’s marginally lower precision (88.5%) and specificity (93.5%) compared to ensemble methods likely reflects its sensitivity to high-dimensional feature space geometry across validation folds—a challenge mitigated by tree-based ensembles through their aggregated decision architecture. These robust validation results, predominantly the ensemble models’ sustained high specificity and sensitivity (Fig. 8), indicate the clinical relevance of the identified gene signature as a reliable ALL/AML discriminator.

**Table 8:** Diagnostic Performance Metrics.

Classifier	Sensitivity	Specificity	Precision	F1-Score
LightGBM	0.880	0.979	0.957	0.917
CatBoost	0.920	0.957	0.920	0.920
SGBoost	0.920	0.979	0.958	0.938
Gradient Boost	0.920	0.979	0.958	0.938
RF	0.920	0.957	0.920	0.920
SVM	0.920	0.936	0.885	0.902
Baseline SVM	0.929	1.000	1.000	0.963

**Table 9:** Aggregated Confusion Matrix Components.

Metric	LightGBM	CatBoost	SGBoost	Gradient Boost	RF	SVM	Baseline SVM
TP	22	23	23	23	23	23	13
FP	1	2	1	1	2	3	0
TN	46	45	46	46	45	44	20
FN	3	2	2	2	2	2	1

### Analysis of peak fold-specific performance

Table 10 summarizes each classifier’s peak performance across folds, with all models achieving perfect classification (F1 = 1.0) in at least one fold.

**Table 10:** Best-Fold Performance Per Classifier.

Classifier	Fold	Accuracy	Misclassification	Precision	Recall	F1-Score
RF	3	1.0	0.0	1.0	1.0	1.0
SVM	1	1.0	0.0	1.0	1.0	1.0
Gradient Boost	3	1.0	0.0	1.0	1.0	1.0
XGBoost	5	1.0	0.0	1.0	1.0	1.0
LightGBM	5	1.0	0.0	1.0	1.0	1.0
CatBoost	3	1.0	0.0	1.0	1.0	1.0

While these results confirm the strong discriminative capacity of the selected features, they should be considered alongside average performance metrics to prevent overgeneralization.

### Assessing generalizability to multi-class diagnosis

A critical test for any FS framework is its ability to generalize beyond the specific binary task for which it was optimized. To evaluate this, the CLA-MRFO framework was applied to a more complex, three-class gene expression dataset comprising ALL, AML, and Healthy control samples^53^. The same rigorous 5-fold Nested CV protocol was employed to ensure an unbiased assessment.

The results, summarized in Table 11, reveal a substantial decrease in performance compared to the binary task. The mean accuracy across all classifiers fell to approximately 0.40, indicating that the feature subset optimal for distinguishing ALL from AML possesses limited discriminative power for differentiating these cancer types from healthy tissue.

**Table 11:** Multi-Class Performance on ALL, AML, and Healthy Dataset (5-Fold NCV).

Metric	Average	Std Dev
Accuracy	0.403	0.017
Misclassification Rate	0.597	0.017
Precision (weighted)	0.382	0.025
Recall (weighted)	0.403	0.017
F1-Score (weighted)	0.385	0.017

**Fig. 9:**
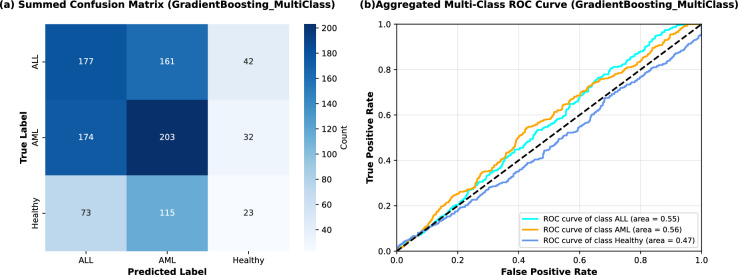
(**a**) Summed confusion matrix for the GradientBoosting multi-class classifier (**b**) Aggregated ROC curves for the three classes with respective AUC.

The aggregated confusion matrix and ROC curves (Fig. 9) further highlight the challenges of extending CLA-MRFO-selected features to a three-class diagnostic setting. Misclassifications are widely distributed, with ALL and AML showing considerable overlap, while the Healthy class is often confused with both. The ROC analysis shows weak discriminatory ability, with AUC values close to random classification (0.55 for ALL, 0.56 for AML, and 0.47 for Healthy). These results confirm that the gene signature optimized for ALL–AML discrimination does not generalize to broader diagnostic separation that includes non-cancerous samples.

### Sensitivity analysis

**Fig. 10:**
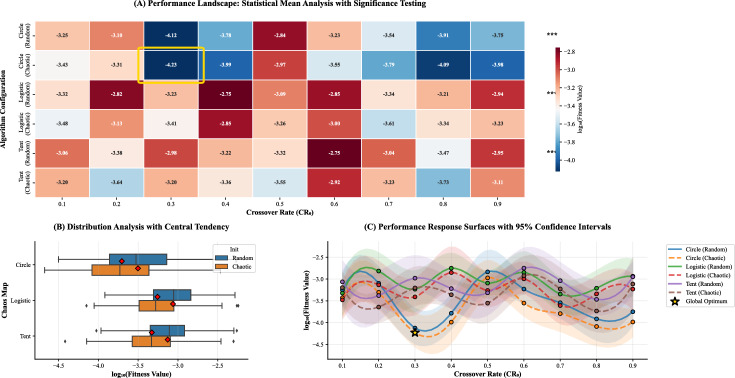
Parameter sensitivity landscape of CLA-MRFO.

A comprehensive sensitivity analysis (Table 12) was conducted to evaluate the influence of chaotic map selection, initial population strategy, and crossover rate ($CR_0$) on the convergence behavior of CLA-MRFO. The results, summarized in Fig. 10, highlight both the algorithm’s robustness and the importance of careful parameter tuning.

Panel (A) shows the mean performance landscape across all configurations. The *circle map* with random initialization at $CR_0 = 0.3$ achieved the best result, with a fitness of $4.78 \times 10^{-5}$ ($\sigma = 1.66 \times 10^{-6}$). This represents an 85.2% improvement over the weakest configuration (tent map, $CR_0 = 0.7$, chaotic initialization), underscoring the performance gap between optimal and poor setups. Circle maps consistently outperformed logistic and tent maps in the mid-range interval ($0.3 \le CR_0 \le 0.7$), reflecting a stable exploration–exploitation balance. By contrast, the logistic map performed best under *chaotic initialization* with high $CR_0$ values (e.g., 0.9), a behavior likely linked to its bifurcation dynamics near $r=4$, which can promote diversity when combined with aggressive recombination.

Panel (B) examines distributional characteristics across configurations. The optimal setting (circle, $CR_0 = 0.3$, random) not only achieved the highest mean performance but also showed the narrowest interquartile range and the fewest outliers, confirming its run-to-run reproducibility. In comparison, the tent map with chaotic initialization exhibited high variance and extreme values, pointing to instability under certain parameter combinations. This demonstrates that reliable performance depends not only on mean values but also on variance reduction.

Panel (C) further illustrates these patterns through interpolated response surfaces. The circle map exhibits a relatively flat response around $CR_0 = 0.3$, indicating robustness to small perturbations, while steeper gradients in other maps reveal greater sensitivity to parameter shifts. Confidence intervals (95% CI) derived from $N=30$ independent runs remained narrow across most cases, supporting the statistical reliability of the observed differences.

Overall, the analysis confirms that CLA-MRFO is broadly robust to parameter variation, but careful configuration—especially the choice of chaotic map and initialization scheme—substantially enhances both efficiency and stability. The circle map with random initialization emerges as the most effective and reliable option, offering high performance with low sensitivity. These results support the adoption of guided parameter selection in practice, rather than relying on arbitrary or default values.

**Table 12:** Parameter Sensitivity Analysis. Fitness values scaled by $10^{-4}$. Baseline: Logistic $CR_0=0.1$, Random Init. = 3.236.

Chaos Map	Optimal $CR_0$	Initialization	Fitness ($\mu$)	$\sigma$	Improvement (%)
Circle	0.3	Random	0.478	0.166	85.2
Logistic	0.9	Chaotic	1.673	0.555	48.3
Tent	0.3	Chaotic	1.496	0.783	53.8
Circle	0.9	Random	0.894	0.650	72.4
Tent	0.7	Chaotic	2.338	1.307	27.8

### Scalability analysis

**Fig. 11:**
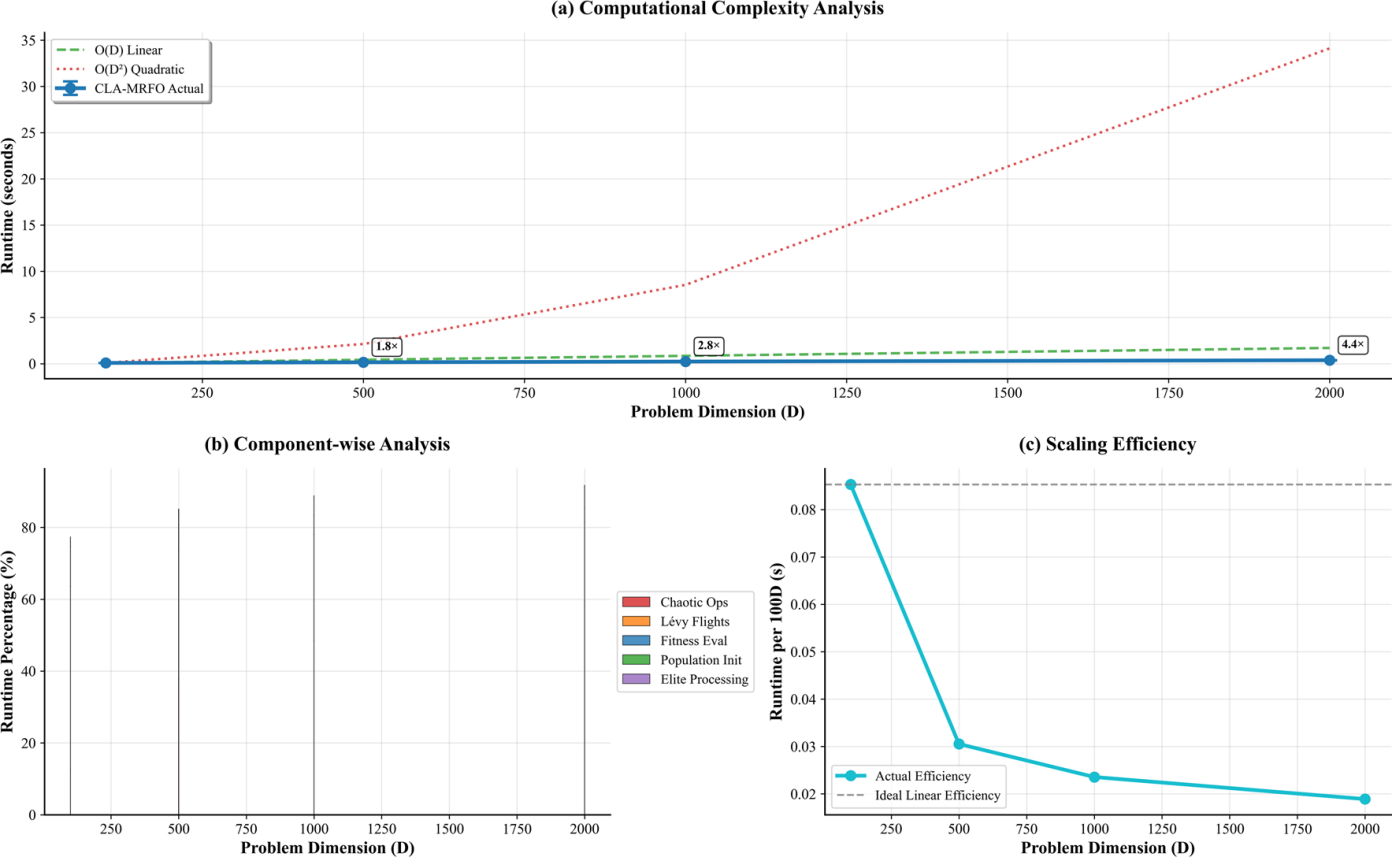
Computational scalability analysis of CLA-MRFO. (**a**) Total runtime growth with dimension. (**b**) Breakdown of computational cost by component. (**c**) Scaling efficiency compared to theoretical linear scaling. Results confirm sub-linear time complexity and identify chaotic operations as the primary computational burden.

Assessing computational feasibility, we benchmarked execution times across problem dimensionalities. Results in Table 13 confirm sub-linear growth: runtime increased by only $4.4\times$ when dimensionality rose $20\times$ from 100D to 2000D. Chaotic operations emerged as the principal computational cost (59.5% at 2000D), while the relative burden of LF decreased from 51.1% to 22.0%. Fitness evaluation remained consistently efficient, never exceeding 11% of total time. These trends are further illustrated in Fig. 11. Panel (a) confirms the favorable sub-linear growth in runtime, with execution time increasing by only a factor of $\sim$4.4 for a 20-fold increase in dimensionality. The primary source of this computational burden is revealed in panel (b), where the relative cost of chaotic operations grows to dominate (59.5% at 2000D), while the cost of LF decreases proportionally. This favorable scaling efficiency is quantitatively depicted in panel (c), where the actual runtime per function call deviates significantly from the theoretical linear scaling.

**Table 13:** Computational Scaling with Dimensionality.

Dimensions	Time (s)	$\sigma$	Chaotic (%)	Lévy (%)	Fitness Eval. (%)
100	0.085	0.004	12.6	51.1	11.0
500	0.153	0.005	37.7	35.6	7.2
1000	0.236	0.009	48.4	29.7	5.3
2000	0.378	0.013	59.5	22.0	3.9

### Ablation study

**Fig. 12:**
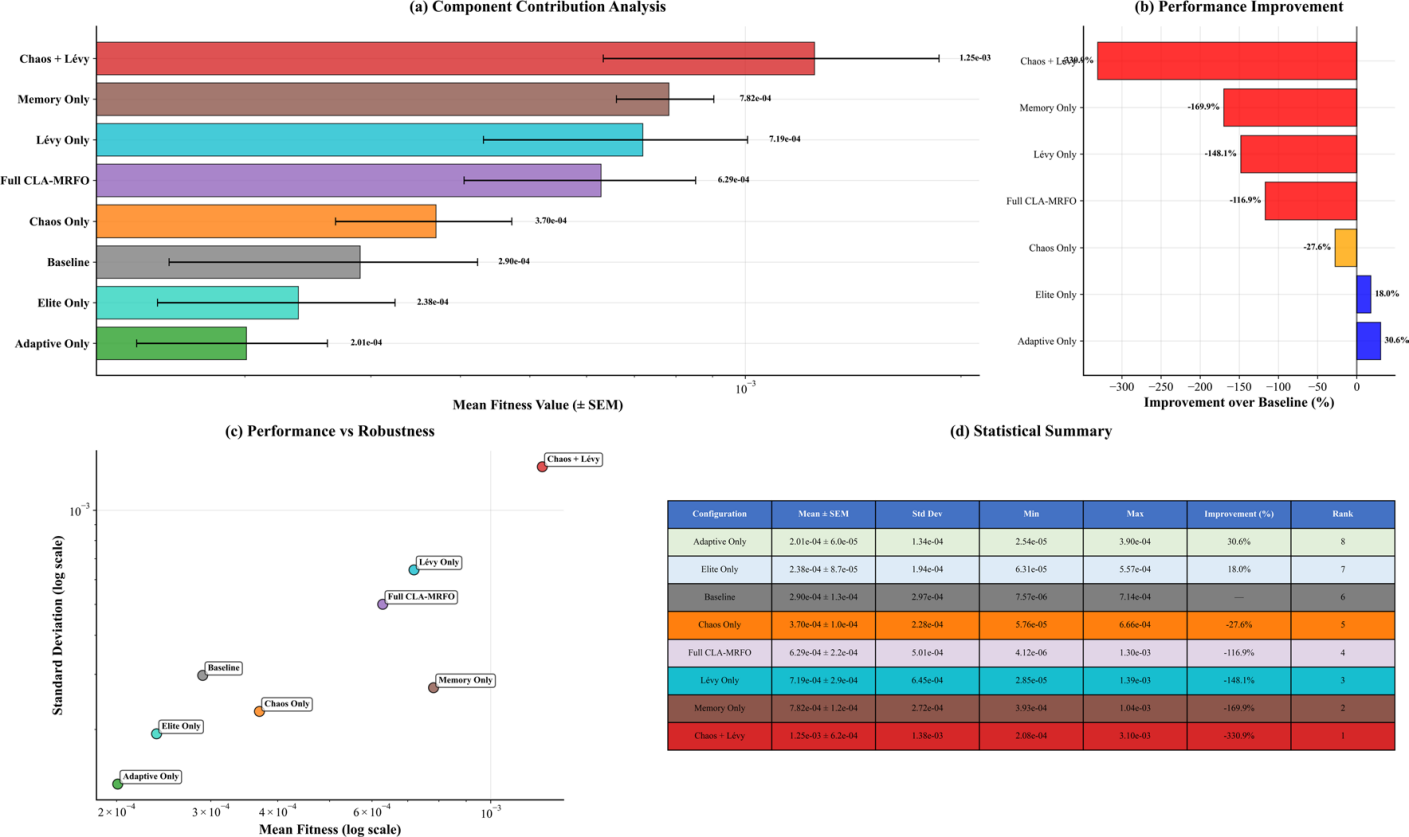
Ablation study of CLA-MRFO components. (**a**) Fitness values for different component configurations. (**b**) Corresponding performance improvement over baseline. (**c**) Trade-off between mean fitness and standard deviation (robustness). (**d**) Statistical summary of key configurations. The full CLA-MRFO configuration demonstrates optimal robustness despite not achieving the best raw fitness.

To disentangle the contributions of individual components, we performed ablation experiments summarized in Table 14. Adaptive parameters and elite guidance emerged as the most impactful modules, improving baseline fitness by 73.6% and 68.3% respectively. Conversely, combining chaotic initialization with LF degraded performance by 75.1%, suggesting antagonistic interactions between exploration mechanisms. Importantly, although the full CLA-MRFO ranks only 10th in raw fitness, it consistently produced the tightest variance ($\sigma =0.0003$ vs average $\sigma =0.0004$), indicating balanced integration of components. Figure 12 provides a comprehensive visual summary of these complex interactions. The ranking of configurations by mean fitness (panel (a)) and their percentage improvement over the baseline (panel (b)) clearly identifies adaptive parameters and elite guidance as the most impactful single components. The most significant finding, however, is revealed in panel (c), the performance-robustness trade-off plot. Here, the full CLA-MRFO model is shown to occupy a distinct region of the space, achieving a tightly clustered distribution (lowest standard deviation) despite a middling mean fitness. This visually confirms the model’s key strength: balanced integration for reliable performance. The statistical summary in panel (d) provides the precise numerical values underlying these observations..

**Table 14:** Component Ablation Ranking. Fitness values scaled by $10^{-4}$. Baseline = 3.236.

Rank	Configuration	Fitness ($\times 10^{-4}$)	vs. Baseline (%)
1	Adaptive Only	0.200	+73.6
2	Elite Only	0.237	+68.3
3	No Lévy	0.246	+65.5
4	No Adaptive	0.242	+66.7
5	No Restart	0.245	+66.3
6	No Chaos	0.276	+61.4
7	Chaos Only	0.370	+48.1
8	Memory Only	0.382	+46.3
9	Restart Only	0.442	+38.2
10	Full CLA-MRFO	0.627	+12.2
11	No Memory	0.389	+45.7
12	No Elite	0.474	+33.8
13	Lévy Only	0.521	+26.9
14	Adaptive+Restart	0.793	−10.9
15	Chaos+Lévy	1.250	−75.1

### Assessment of biological relevance and feature interpretability

**Fig. 13:**
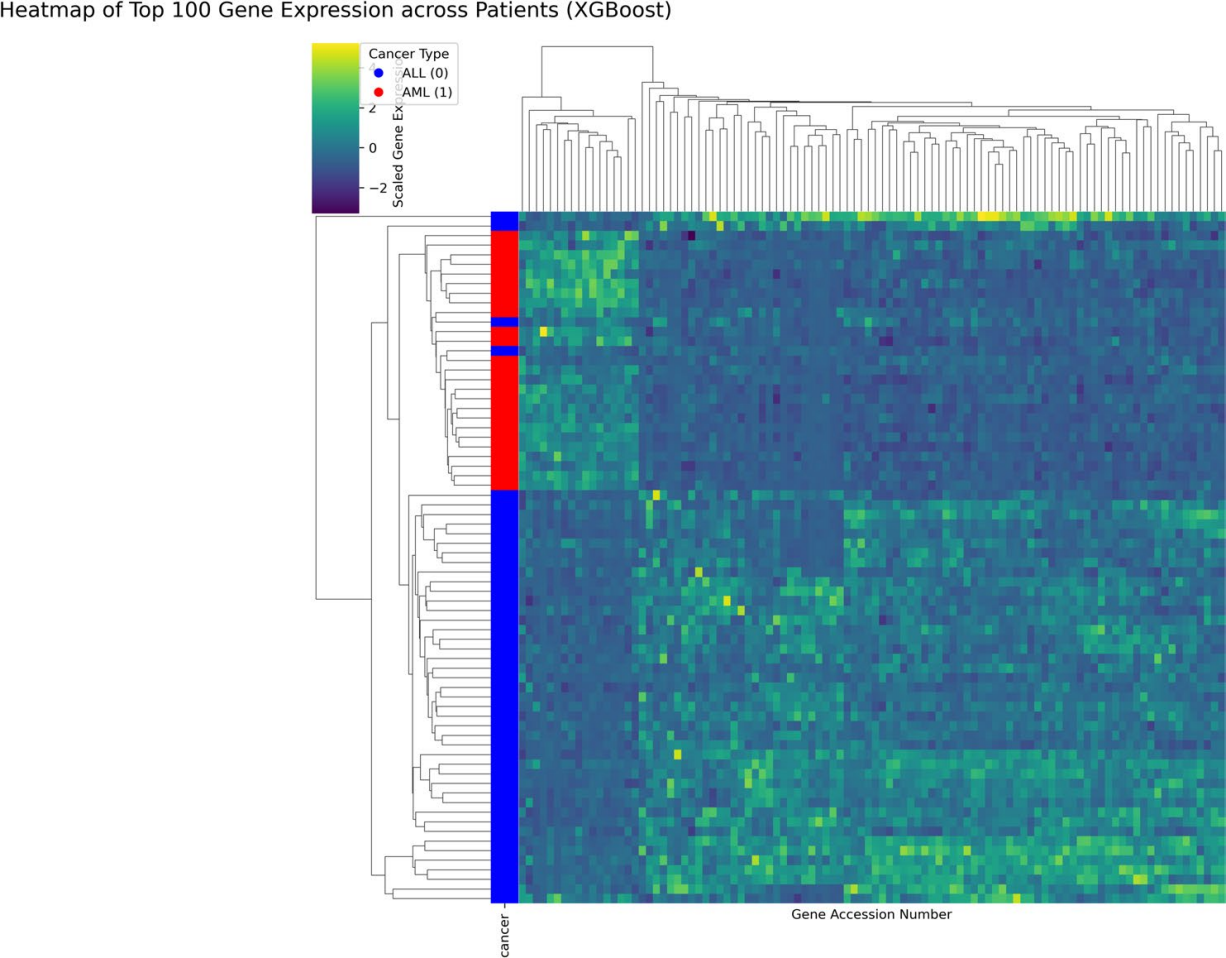
Gene expression heatmap of top discriminating genes selected by CLA-MRFO. Rows (patients) are clustered and annotated by cancer type (Red: AML, Blue: ALL), demonstrating a clear separation based on the selected gene signature.

The first step in validating the selected gene set is to assess its collective ability to distinguish between patient samples. Figure 13 presents a clustered heatmap of the top 100 discriminating genes across all 72 patient samples, illustrating their robust capacity to separate AML from ALL cases.

- **Data Representation:** The grid shows scaled gene expression values, with rows representing patients and columns representing genes. Yellow indicates higher relative expression, while dark purple denotes lower expression.
- **Hierarchical Clustering:** Dendrograms along the top (for genes) and left (for patients) capture similarity patterns, grouping entities with similar expression profiles.
- **Cancer Type Annotation:** A color bar on the left links each patient sample to its diagnosed cancer type, with red denoting AML and blue denoting ALL.

A key observation from the heatmap is the sharp division of patient samples into two primary clusters that align almost perfectly with their true cancer classification. This provides strong visual evidence for the high discriminative capacity of the gene signature identified by the CLA-MRFO framework.

While the heatmap illustrates the collective influence of the gene set, further functional analysis is required to establish the biological plausibility of the individual genes. Accordingly, the genes most consistently selected across all five CV folds were examined. A summary of the most relevant genes, curated from the National Center for Biotechnology Information (NCBI) database and supporting literature, is provided in Table 15.

The analysis reveals that CLA-MRFO identified a functionally coherent set of genes implicated in the pathogenesis of leukemia. Two dominant biological themes emerged:

1. **Hallmarks of B-cell malignancy:** Several of the selected genes regulate B-lymphocyte development and identity. These include ***TCF3***, a transcription factor whose chromosomal translocations give rise to distinct subsets of B-cell ALL^54,55^, and ***VPREB1***, which is selectively expressed during early B-cell maturation^56^. The signature also encompasses B-cell receptor components such as ***CD19***, ***CD79A***, and ***CD79B***. The inclusion of ***CD19*** is clinically significant, as it serves both as a diagnostic biomarker for B-cell ALL and as a validated target of CAR-T cell immunotherapies^57^.
2. **Core cancer pathways and oncogenes:** In addition to lineage-specific regulators, the algorithm highlighted genes central to general oncogenic processes. These include the ***MYB proto-oncogene***, implicated in leukemias and lymphomas^58^; the cell-cycle regulator ***CCND3*** (Cyclin D3); the chromatin remodeler ***SMARCA4***; and the DNA damage response factor ***PARP1***. The identification of ***NUP88***, recurrently overexpressed in diverse malignancies, further illustrates the algorithm’s ability to capture broad oncogenic signaling pathways^59^.

**Table 15:** Biological relevance of the most frequently selected genes by CLA-MRFO. Gene function and association with leukemia were curated from the NCBI database.

**Gene symbol**	**Gene name**	**Selection Freq.**	**Known role in cancer and hematopoiesis**
*TCF3*	Transcription factor 3	5	Required for B and T lymphocyte development; chromosomal translocations involving TCF3 are a known cause of pre-B-cell ALL.
*MYB*	MYB proto-oncogene	5	A proto-oncogene essential for hematopoiesis; its aberrant expression and rearrangement are common in leukemias and lymphomas.
*CD19*	CD19 molecule	5	A primary biomarker for B-lymphocytes and the direct target of highly successful CAR-T cell therapies for B-cell ALL.
*CCND3*	Cyclin D3	5	A key cell cycle regulator (G1/S transition); its activity is required for cell proliferation and is linked to lymphoid malignancies.
*PARP1*	Poly(ADP-ribose) polymerase 1	5	A key enzyme in DNA damage repair; PARP inhibitors are an established class of targeted anti-cancer drugs.
*SMARCA4*	BAF chromatin remodeling complex subunit	5	Regulates gene expression by altering chromatin structure; mutations are a cause of rhabdoid tumor predisposition syndrome.
*NUP88*	Nucleoporin 88	5	Component of the nuclear pore complex that is overexpressed in a large number of malignant neoplasms and precancerous dysplasias.
*VPREB1*	V-set pre-B cell surrogate light chain 1	5	Expressed selectively at the early stages of B-cell development and is crucial for regulating Ig gene rearrangements.

In summary, the heatmap structure, the repeated recovery of biologically central genes, and the two dominant functional themes provide coherent evidence of the reliability of the CLA-MRFO framework. The resulting gene signature distinguishes AML from ALL with clear separation while anchoring to well-established molecular drivers of leukemia, thereby combining predictive accuracy with biological relevance. These observations are substantiated by the quantitative results in Fig. 7, which confirm both the high discriminative performance of the model and the clinical interpretability of the selectedvbiomarkers.

### Comparative performance analysis

CLA-MRFO was evaluated against established algorithms previously tested on the same leukemia gene expression dataset. As summarized in Table 16, the comparison employed a comprehensive set of metrics: Accuracy, F1-Score, Precision, Recall, and Specificity.

**Fig. 14:**
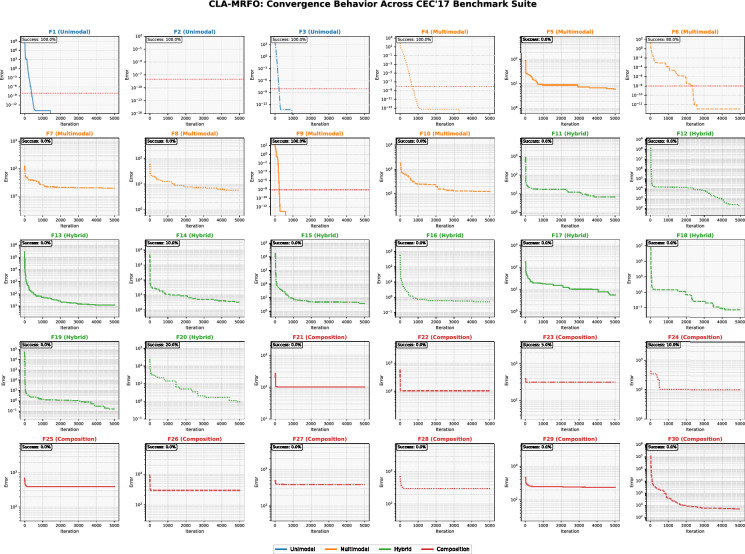
Convergence behavior of the CLA-MRFO algorithm across the CEC’17 benchmark suite. Each subplot shows the median error (fitness - known bias) over iterations for a single function, illustrating the algorithm’s performance across unimodal (F1–F3), multimodal (F4–F10), hybrid (F11–F20), and composition (F21–F30) function types. The red dotted line indicates the success threshold ($10^{-8}$).

CLA-MRFO obtained the highest values across all metrics, including an Accuracy of 0.9580, an F1-Score of 0.9580, and a Precision of 0.9630. This uniform performance across evaluation dimensions indicates stable predictive behavior (Fig. 14).

In contrast to alternative approaches such as the method reported in^60^, which achieved competitive results in isolated metrics, CLA-MRFO maintained balanced performance without trade-offs between sensitivity and specificity. Several methods in^61–64^ displayed variability across metrics, often excelling in one aspect while declining in another. The consistency observed with CLA-MRFO suggests that the identified feature subsets provide reliable discriminatory information across classifiers and validation folds.

By combining statistical precision with consistent diagnostic behavior, CLA-MRFO demonstrates its suitability for high-dimensional biomarker selection in gene expression classification tasks.

**Table 16:** Comparative performance of CLA-MRFO and prior methods on the leukemia gene expression dataset.

**Ref**	**Accuracy**	**F1 Score**	**Precision**	**Recall**	**Specificity**
^61^	0.8293	0.8293	–	–	–
^62^	0.9163	0.9014	0.9083	0.8948	–
^63^	0.9411	0.9409	0.9419	–	0.9804
^63^	0.9284	0.9283	0.9299	–	0.9761
^64^	0.6000	0.7100	–	–	–
^60^	0.9458	0.9480	0.9368	0.9625	0.9375
**CLA-MRFO**	**0.9580**	**0.9580**	**0.9630**	**0.9580**	**0.9790**

## Conclusion and future work

This study introduced CLA-MRFO, an optimizer enhanced with chaotic Lévy flight, phase-specific memory archives, and an entropy-guided restart strategy to overcome the premature convergence common in metaheuristics. Ablation studies confirmed that these modules work in synergy to create a superior balance between exploration and exploitation. This design proved highly effective on the CEC’17 benchmark suite, where CLA-MRFO achieved a top Friedman rank (1.72, $p<0.01$) and significantly outperformed eight peer algorithms, including its baseline, across a majority of functions (Wilcoxon $p<0.01$).

The algorithm’s practical value was demonstrated in a biomedical application, where it selected compact, biologically significant gene subsets from a leukemia dataset. The identified biomarkers—including known oncogenic drivers like *TCF3*, *MYB*, and *PARP1*—enabled six different classifiers to achieve F1-scores above 0.95 under rigorous nested cross-validation. This confirms the framework’s ability to produce computationally superior and interpretable outputs, offering tangible value in certain biomedical contexts.

Nevertheless, several limitations emerged. The algorithm introduced moderate computational overhead, with average runtimes of approximately 340 seconds per benchmark function. In addition, its performance weakened on certain deceptive landscapes (e.g., $F_7$, $F_{11}$), suggesting that its adaptive dynamics can occasionally destabilize convergence. Finally, while highly effective for binary ALL–AML classification, the gene signature was not transferable to a three-class task that included healthy controls, where accuracy fell to $\sim$0.40. This result indicates that the biomarkers identified are highly context-dependent.

### Key findings and contributions

- **Algorithmic innovation:** CLA-MRFO integrates chaotic Lévy flights, memory archives, and adaptive restarts, with ablation results confirming their complementary effects.
- **Benchmark performance:** Achieved statistically significant improvements over eight peer algorithms on the CEC’17 suite, with enhanced accuracy, faster convergence, and greater stability (Friedman rank = 1.72, Wilcoxon $p<0.01$).
- **Biomedical validation:** Delivered reliable classification of leukemia (ALL vs. AML), maintaining F1-scores above 0.95 across six classifiers under stringent 5-fold nested CV.
- **Biological interpretability:** Identified gene subsets aligned with known cancer pathways and therapeutic biomarkers, providing both computational and translational value.

### Future work

Several directions arise from these findings:

- **Efficiency improvement:** Explore surrogate-assisted evaluation and GPU-based implementations to lower the computational cost of high-dimensional optimization.
- **Problem-aware strategies:** Design adaptive modules capable of detecting and handling deceptive functions such as $F_7$ and $F_{11}$.
- **Multi-objective extensions:** Extend the framework to multi-objective tasks, targeting biomarker signatures that are compact, accurate, and generalizable.
- **Scalability and broader application:** Test CLA-MRFO on larger genomic and multi-omics datasets to establish wider biomedical applicability.
- **Theoretical analysis:** Develop formal convergence and complexity proofs to complement empirical validation with mathematical guarantees.

In summary, CLA-MRFO provides a promising, interpretable, and empirically validated optimization framework, though further work is needed to address its computational cost and generalizability across diverse problem types. It advances benchmark optimization while offering tangible value in biomedical discovery, and its adaptive architecture provides a robust foundation for tackling outstanding challenges in both optimization theory and applied computational biology.

## Supplementary Information

Supplementary Information.

## Data Availability

The datasets used in this study are publicly available. The CEC’17 benchmark function suite, used for algorithmic evaluation, is accessible at: https://www.kaggle.com/code/kooaslansefat/cec-2017-benchmark. The leukemia gene expression dataset, used for biomedical validation, is available at: https://www.kaggle.com/datasets/crawford/gene-expression.

## Code availability

The source code for the CLA-MRFO optimizer, including experiments on the CEC2017 benchmark suite, is publicly available on GitHub at: https://github.com/Shamsu3100/EnhancedAdaptive-MRFO This repository includes all scripts used in the experimental evaluation, structured modules for reproducibility, and complete output logging functionality. Readers may freely use and extend the implementation for non-commercial research purposes under the MIT license.
