# Supplementary material for: Chaotic Lévy and adaptive restart enhance the Manta Ray foraging optimizer for gene feature selection
Source: Sci Rep. 2025 Nov 25;15:41930. doi: 10.1038/s41598-025-25766-y (PMC12647646; doi:10.1038/s41598-025-25766-y)
Supplement: Supplementary file 1 — Supplementary Information. [file 41598_2025_25766_MOESM1_ESM.pdf]

Appendix A : Benchmark Results Tables

Table A.1: CLA-MRFO Results on CEC'17

Func	Category	Best	Median	Success Rate (%)	Avg Conv. Iter	Variance	IQR	Runtime (s)	FuncEvals
1	Unimodal	8.53×10^{-15}	0.00	100.0	214.15	1.29×10^{-28}	1.42×10^{-14}	261.69	2 081 616.20
2	Unimodal	0.00	0.00	100.0	1.00	0.00	0.00	214.04	2 073 670.90
3	Unimodal	8.53×10^{-15}	0.00	100.0	126.55	4.12×10^{-28}	0.00	249.15	2 072 086.60
4	Multimodal	3.13×10^{-14}	5.68×10^{-14}	100.0	262.80	8.00×10^{-28}	5.68×10^{-14}	265.95	2 059 730.60
5	Multimodal	5.83	5.47	0.0	5000.00	2.36	1.99	354.21	2 074 346.50
6	Multimodal	3.03×10^{-7}	1.14×10^{-13}	100.0	172.20	1.53×10^{-12}	1.14×10^{-13}	255.08	2 037 058.70
7	Multimodal	1.96×10^{1}	2.02×10^{1}	0.0	5000.00	3.37×10^{1}	7.99	341.29	2 076 485.00
8	Multimodal	6.52	6.96	0.0	5000.00	2.82	2.24	335.34	2 073 629.55
9	Multimodal	3.98×10^{-14}	0.00	100.0	241.10	2.94×10^{-27}	1.14×10^{-13}	263.40	2 063 044.75
10	Multimodal	7.95×10^{1}	8.60×10^{1}	0.0	5000.00	3.98×10^{3}	1.25×10^{2}	375.78	2 045 110.95
11	Hybrid	1.05×10^{1}	1.08×10^{1}	0.0	5000.00	4.70×10^{1}	8.03	497.28	2 083 702.70
12	Hybrid	9.44×10^{2}	2.47×10^{2}	0.0	5000.00	2.82×10^{6}	5.79×10^{2}	496.75	2 082 316.55
13	Hybrid	1.28×10^{1}	1.42×10^{1}	5.0	4934.50	4.24×10^{1}	1.00×10^{1}	503.18	2 079 892.95
14	Hybrid	3.46	1.99	5.0	4993.25	9.83	3.93	337.64	2 051 581.80
15	Hybrid	3.69	2.01	0.0	5000.00	1.54×10^{1}	4.11	514.60	2 092 335.35
16	Hybrid	4.50×10^{-1}	4.62×10^{-1}	5.0	4939.05	5.58×10^{-2}	2.71×10^{-1}	291.48	2 025 687.10
17	Hybrid	7.07	3.86	0.0	5000.00	5.48×10^{1}	9.87	296.32	2 030 055.10
18	Hybrid	4.29×10^{-1}	1.30×10^{-1}	35.0	4199.05	3.09×10^{-1}	9.95×10^{-1}	308.63	2 042 629.35
19	Hybrid	4.28×10^{-1}	1.77×10^{-1}	0.0	5000.00	2.02×10^{-1}	9.60×10^{-1}	329.80	2 028 927.15
20	Hybrid	2.22	9.95×10^{-1}	35.0	4360.65	7.88	3.98	304.02	2 045 992.80
21	Composition	9.60×10^{1}	1.00×10^{2}	5.0	4817.30	4.86×10^{2}	2.62	293.82	2 048 382.20
22	Composition	9.92×10^{1}	1.02×10^{2}	0.0	5000.00	2.29×10^{2}	1.51	317.72	2 050 044.75
23	Composition	2.99×10^{2}	3.13×10^{2}	5.0	4861.50	4.76×10^{3}	7.32	314.69	2 047 756.80
24	Composition	1.00×10^{2}	1.00×10^{2}	0.0	5000.00	5.12×10^{-26}	4.55×10^{-13}	299.63	2 041 286.85
25	Composition	3.68×10^{2}	3.98×10^{2}	0.0	5000.00	7.96×10^{3}	3.70×10^{-2}	424.47	2 090 210.50
26	Composition	2.92×10^{2}	3.00×10^{2}	0.0	5000.00	2.49×10^{3}	1.18×10^{1}	315.58	2 043 391.25
27	Composition	3.89×10^{2}	3.89×10^{2}	0.0	5000.00	6.79×10^{-1}	1.22	438.22	2 090 212.10
28	Composition	3.11×10^{2}	3.00×10^{2}	0.0	5000.00	1.05×10^{3}	0.00	308.03	2 042 550.65
29	Composition	2.38×10^{2}	2.38×10^{2}	0.0	5000.00	1.09×10^{1}	4.47	452.41	2 100 129.95
30	Composition	5.63×10^{2}	5.41×10^{2}	0.0	5000.00	1.90×10^{4}	1.13×10^{2}	479.88	2 087 996.60

Table A.2: Comparison of MRFO and CLA-MRFO Across CEC'17 Functions

Func	Category	MRFO				CLA-MRFO			
		Mean	SD	Conv. Iter	Success (%)	Mean	SD	Conv. Iter	Success (%)
1	Unimodal	514.98	671.74	5000.00	0.0	**8.53e-15**	**1.14e-14**	**214.15**	**100.0**
2	Unimodal	0.00	0.00	1.00	100.0	0.00	0.00	1.00	100.0
3	Unimodal	0.00	0.00	378.85	100.0	**8.53e-15**	**2.03e-14**	**126.55**	100.0
4	Multimodal	0.17	0.13	4510.00	15.0	**3.13e-14**	**2.83e-14**	**262.80**	**100.0**
5	Multimodal	8.36	5.05	5000.00	0.0	**5.83**	**1.54**	5000.00	0.0
6	Multimodal	5.67	2.47	**112.75**	100.0	**3.03e-07**	3.91	172.20	100.0
7	Multimodal	**16.41**	**3.76**	5000.00	0.0	19.56	5.81	5000.00	0.0
8	Multimodal	7.17	4.01	5000.00	0.0	**6.52**	**1.68**	5000.00	0.0
9	Multimodal	0.02	0.10	331.35	95.0	**3.98e-14**	**5.42e-14**	**241.10**	**100.0**
10	Multimodal	186.37	128.48	5000.00	0.0	**79.53**	**63.11**	5000.00	0.0
11	Hybrid	**4.06**	**3.07**	**4808.30**	**5.0**	10.53	6.86	5000.00	0.0
12	Hybrid	11 869.53	7749.98	5000.00	0.0	**944.18**	**1679.24**	5000.00	0.0
13	Hybrid	154.32	99.07	5000.00	0.0	**12.82**	**6.52**	**4934.50**	**5.0**
14	Hybrid	6.46	4.42	5000.00	0.0	**3.46**	**3.14**	**4993.25**	**5.0**
15	Hybrid	3.90	2.78	5000.00	0.0	**3.69**	3.93	5000.00	0.0
16	Hybrid	2.31	8.30	5000.00	0.0	**0.45**	**0.24**	**4939.05**	**5.0**
17	Hybrid	13.29	13.18	5000.00	0.0	**7.07**	**7.40**	5000.00	0.0
18	Hybrid	744.38	738.24	5000.00	0.0	**0.43**	**0.56**	**4199.05**	**35.0**
19	Hybrid	3.93	1.71	5000.00	0.0	**0.43**	**0.45**	5000.00	0.0
20	Hybrid	2.99	4.48	**4767.95**	**5.0**	**2.22**	2.81	4360.65	**35.0**
21	Composition	100.26	**0.78**	5000.00	0.0	**95.96**	22.04	**4817.30**	**5.0**
22	Composition	**97.08**	19.66	5000.00	0.0	99.24	**15.13**	5000.00	0.0
23	Composition	311.96	**3.74**	5000.00	0.0	**298.74**	68.98	**4861.50**	**5.0**
24	Composition	100.00	**6.86e-13**	5000.00	0.0	100.00	7.16	5000.00	0.0
25	Composition	421.81	23.08	5000.00	0.0	**368.07**	**89.22**	5000.00	0.0
26	Composition	299.82	31.07	5000.00	0.0	**291.79**	**49.88**	5000.00	0.0
27	Composition	395.81	3.08	5000.00	0.0	**388.71**	**0.82**	5000.00	0.0
28	Composition	**300.00**	0.00	5000.00	0.0	310.76	**32.45**	5000.00	0.0
29	Composition	247.97	10.07	5000.00	0.0	**238.23**	**3.30**	5000.00	0.0
30	Composition	1421.52	543.42	5000.00	0.0	**563.01**	**137.92**	5000.00	0.0

Table A.3: Performance comparison of different algorithms (Mean and STD values)

F	Measure	MFO	WOA	SCA	HHO	BWO	MRFO	GBO	MRFO-GBO	CLA-MRFO
F1	Mean	2.45×10^{3}	1.73×10^{3}	3.14×10^{3}	1.50×10^{3}	1.73×10^{3}	6.05×10^{2}	8.74×10^{2}	6.16×10^{2}	**1.00E+02**
	STD	5.77×10^{2}	6.83×10^{1}	1.72×10^{2}	2.33	7.49×10^{1}	5.67	1.50×10^{2}	1.19×10^{1}	**1.14E-14**
F2	Mean	-	-	-	-	-	-	-	-	**0.00E+00**
	STD	-	-	-	-	-	-	-	-	**0.00E+00**
F3	Mean	1.86×10^{3}	1.26×10^{3}	1.83×10^{3}	1.35×10^{3}	1.43×10^{3}	1.06×10^{3}	6.93×10^{2}	5.28×10^{2}	**3.00E+02**
	STD	4.49×10^{2}	4.03×10^{2}	2.63×10^{2}	2.37×10^{2}	2.01×10^{2}	1.56×10^{2}	1.43×10^{2}	1.44×10^{2}	**2.03E-14**
F4	Mean	7.18×10^{3}	4.64×10^{3}	3.36×10^{3}	2.72×10^{3}	2.78×10^{3}	2.14×10^{3}	1.36×10^{3}	1.01×10^{3}	**4.00E+02**
	STD	1.59×10^{3}	1.15×10^{3}	4.82×10^{2}	2.84×10^{2}	3.82×10^{2}	4.00×10^{2}	2.66×10^{2}	2.39×10^{2}	**2.83E-14**
F5	Mean	1.01×10^{3}	1.06×10^{3}	1.11×10^{3}	9.16×10^{2}	7.60×10^{2}	8.27×10^{2}	8.12×10^{2}	6.80×10^{2}	**5.06E+02**
	STD	1.07×10^{2}	7.81×10^{1}	3.17×10^{1}	4.12×10^{1}	3.34×10^{1}	4.58×10^{1}	5.33×10^{1}	3.62×10^{1}	**1.54E+00**
F6	Mean	6.57×10^{2}	6.92×10^{2}	6.82×10^{2}	6.77×10^{2}	6.37×10^{2}	6.51×10^{2}	6.40×10^{2}	6.24×10^{2}	**6.00E+02**
	STD	1.03×10^{1}	1.18×10^{1}	6.79	4.19	4.30	7.45	9.50	8.03	**1.24E-06**
F7	Mean	2.23×10^{3}	1.84×10^{3}	1.77×10^{3}	1.82×10^{3}	1.15×10^{3}	1.51×10^{3}	1.25×10^{3}	1.01×10^{3}	**7.20E+02**
	STD	4.53×10^{2}	8.72×10^{1}	7.72×10^{1}	1.05×10^{2}	4.82×10^{1}	1.85×10^{2}	9.78×10^{1}	7.73×10^{1}	**5.81E+00**
F8	Mean	1.28×10^{3}	1.33×10^{3}	1.42×10^{3}	1.20×10^{3}	1.07×10^{3}	1.15×10^{3}	1.13×10^{3}	1.03×10^{3}	**8.07E+02**
	STD	8.85×10^{1}	5.28×10^{1}	5.26×10^{1}	3.31×10^{1}	2.99×10^{1}	4.69×10^{1}	4.48×10^{1}	5.57×10^{1}	**1.68E+00**
F9	Mean	4.07×10^{3}	7.16×10^{3}	6.22×10^{3}	6.12×10^{3}	1.96×10^{3}	2.29×10^{3}	1.73×10^{3}	1.01×10^{3}	**9.00E+02**
	STD	1.28×10^{3}	1.67×10^{3}	8.89×10^{2}	5.31×10^{2}	4.79×10^{2}	4.34×10^{2}	5.46×10^{2}	3.64×10^{2}	**1.71E-13**
F10	Mean	1.39×10^{3}	2.06×10^{3}	2.54×10^{3}	1.66×10^{3}	1.38×10^{3}	1.26×10^{3}	1.33×10^{3}	1.22×10^{3}	**1.08E+03**
	STD	1.33×10^{2}	1.87×10^{2}	6.85×10^{1}	1.50×10^{2}	1.14×10^{2}	1.23×10^{2}	1.58×10^{2}	1.14×10^{2}	**6.31E+01**
F11	Mean	1.74×10^{4}	5.35×10^{3}	1.24×10^{4}	1.75×10^{3}	5.99×10^{3}	1.26×10^{3}	1.39×10^{3}	1.29×10^{3}	**1.11E+03**
	STD	1.71×10^{4}	1.37×10^{3}	2.08×10^{3}	1.52×10^{2}	2.14×10^{3}	3.59×10^{1}	8.32×10^{1}	3.95×10^{1}	**6.85E+00**
F12	Mean	5.71×10^{6}	2.09×10^{6}	1.90×10^{7}	2.22×10^{5}	3.61×10^{6}	**2.29E+03**	8.24×10^{3}	1.73×10^{3}	2.14×10^{3}
	STD	4.92×10^{6}	1.18×10^{6}	4.59×10^{6}	1.77×10^{5}	1.75×10^{6}	1.30×10^{3}	1.14×10^{4}	**1.29E+03**	1.68×10^{3}
F13	Mean	5.48×10^{8}	5.97×10^{7}	1.81×10^{9}	2.65×10^{6}	4.30×10^{8}	2.46×10^{3}	5.41×10^{3}	**1.91E+03**	1.31×10^{3}
	STD	6.06×10^{8}	6.95×10^{7}	9.18×10^{8}	4.89×10^{6}	2.92×10^{8}	2.21×10^{3}	3.73×10^{3}	1.46×10^{3}	**6.52E+00**
F14	Mean	5.76×10^{4}	2.07×10^{5}	2.81×10^{5}	1.14×10^{5}	4.75×10^{5}	4.05×10^{3}	3.08×10^{3}	1.98×10^{3}	**1.40E+03**
	STD	4.96×10^{4}	1.28×10^{5}	1.18×10^{5}	8.13×10^{4}	3.15×10^{5}	3.06×10^{3}	2.73×10^{3}	1.33×10^{3}	**3.14E+00**
F15	Mean	1.84×10^{8}	1.68×10^{7}	8.95×10^{8}	9.43×10^{5}	1.73×10^{8}	9.62×10^{3}	1.59×10^{4}	7.01×10^{3}	**1.50E+03**
	STD	6.40×10^{8}	1.51×10^{7}	2.86×10^{8}	3.18×10^{5}	1.21×10^{8}	5.60×10^{3}	9.32×10^{3}	5.24×10^{3}	**3.93E+00**
F16	Mean	4.34×10^{3}	3.38×10^{3}	6.26×10^{3}	6.15×10^{3}	4.48×10^{3}	3.57×10^{3}	3.41×10^{3}	3.23×10^{3}	**1.60E+03**
	STD	5.83×10^{2}	4.00×10^{2}	8.69×10^{2}	2.97×10^{2}	6.50×10^{2}	4.31×10^{2}	4.13×10^{2}	3.12×10^{2}	**7.47E-01**
F17	Mean	3.96×10^{3}	4.31×10^{3}	4.75×10^{3}	3.91×10^{3}	3.15×10^{3}	3.31×10^{3}	3.29×10^{3}	3.16×10^{3}	**1.71E+03**
	STD	6.16×10^{2}	6.17×10^{2}	2.84×10^{2}	4.38×10^{2}	2.05×10^{2}	4.17×10^{2}	3.32×10^{2}	3.65×10^{2}	**7.40E+00**
F18	Mean	1.06×10^{4}	6.54×10^{4}	4.72×10^{4}	1.29×10^{4}	1.56×10^{4}	7.31×10^{3}	3.15×10^{3}	3.06×10^{3}	**1.80E+03**
	STD	1.90×10^{4}	4.26×10^{4}	3.01×10^{4}	1.54×10^{4}	9.12×10^{3}	5.64×10^{3}	2.42×10^{3}	2.09×10^{3}	**5.56E-01**
F19	Mean	3.11×10^{4}	2.17×10^{4}	4.76×10^{4}	2.00×10^{4}	2.36×10^{4}	9.75×10^{3}	7.37×10^{3}	7.30×10^{3}	**1.90E+03**
	STD	6.51×10^{4}	2.53×10^{4}	2.48×10^{4}	1.55×10^{4}	2.81×10^{4}	5.13×10^{3}	6.20×10^{3}	3.86×10^{3}	**4.49E-01**
F20	Mean	3.74×10^{3}	3.96×10^{3}	4.25×10^{3}	3.47×10^{3}	3.11×10^{3}	3.18×10^{3}	3.23×10^{3}	3.18×10^{3}	**2.00E+03**
	STD	3.26×10^{2}	3.64×10^{2}	2.16×10^{2}	2.81×10^{2}	3.27×10^{2}	4.35×10^{2}	3.36×10^{2}	2.68×10^{2}	**2.81E+00**
F21	Mean	2.80×10^{3}	3.03×10^{3}	2.94×10^{3}	2.89×10^{3}	2.58×10^{3}	2.58×10^{3}	2.58×10^{3}	2.48×10^{3}	**2.20E+03**
	STD	6.83×10^{1}	1.23×10^{2}	5.27×10^{1}	6.70×10^{1}	2.99×10^{1}	6.22×10^{1}	5.04×10^{1}	4.87×10^{1}	**2.20E+01**
F22	Mean	1.08×10^{4}	1.46×10^{4}	1.69×10^{4}	1.15×10^{4}	9.69×10^{3}	9.64×10^{3}	9.35×10^{3}	9.09×10^{3}	**2.30E+03**
	STD	9.86×10^{2}	1.05×10^{3}	3.39×10^{2}	8.12×10^{2}	1.29×10^{3}	2.06×10^{3}	2.54×10^{3}	1.10×10^{3}	**1.51E+01**
F23	Mean	3.17×10^{3}	3.79×10^{3}	3.62×10^{3}	4.00×10^{3}	3.45×10^{3}	3.15×10^{3}	3.10×10^{3}	3.01×10^{3}	**2.30E+03**
	STD	6.63×10^{1}	1.53×10^{2}	8.07×10^{1}	2.32×10^{2}	7.07×10^{1}	9.49×10^{1}	8.79×10^{1}	6.16×10^{1}	**6.90E+01**
F24	Mean	3.26×10^{3}	3.85×10^{3}	3.83×10^{3}	4.39×10^{3}	3.76×10^{3}	3.33×10^{3}	3.24×10^{3}	3.13×10^{3}	**2.50E+03**
	STD	7.95×10^{1}	1.52×10^{2}	7.68×10^{1}	2.45×10^{2}	8.95×10^{1}	9.93×10^{1}	9.53×10^{1}	6.15×10^{1}	**7.16E-13**
F25	Mean	5.22×10^{3}	4.24×10^{3}	7.83×10^{3}	3.27×10^{3}	4.22×10^{3}	3.09×10^{3}	3.11×10^{3}	3.09×10^{3}	**2.87E+03**
	STD	1.73×10^{3}	4.52×10^{2}	7.98×10^{2}	6.64×10^{1}	3.39×10^{2}	2.53×10^{1}	3.04×10^{1}	2.42×10^{1}	**8.92E+01**
F26	Mean	8.98×10^{3}	1.46×10^{4}	1.34×10^{4}	1.16×10^{4}	1.05×10^{4}	9.57×10^{3}	8.32×10^{3}	7.44×10^{3}	**2.92E+03**
	STD	1.15×10^{3}	1.43×10^{3}	7.12×10^{2}	7.20×10^{2}	9.10×10^{2}	3.02×10^{3}	2.48×10^{3}	1.17×10^{3}	**4.99E+01**
F27	Mean	2.84×10^{3}	3.60×10^{3}	3.69×10^{3}	3.66×10^{3}	3.63×10^{3}	2.92×10^{3}	2.84×10^{3}	2.80×10^{3}	**3.09E+03**
	STD	9.78×10^{1}	4.12×10^{2}	1.92×10^{2}	5.02×10^{2}	1.97×10^{2}	1.33×10^{2}	1.18×10^{2}	**6.09E+01**	8.24×10^{-1}
F28	Mean	8.44×10^{3}	5.16×10^{3}	8.05×10^{3}	3.82×10^{3}	4.44×10^{3}	3.35×10^{3}	3.38×10^{3}	3.37×10^{3}	**3.11E+03**
	STD	1.01×10^{3}	4.30×10^{2}	7.28×10^{2}	1.55×10^{2}	3.41×10^{2}	3.00×10^{1}	3.04×10^{1}	3.05×10^{1}	**3.25E+01**
F29	Mean	5.37×10^{3}	5.44×10^{3}	8.75×10^{3}	8.86×10^{3}	6.61×10^{3}	4.80×10^{3}	4.67×10^{3}	4.47×10^{3}	**3.24E+03**
	STD	6.04×10^{2}	4.81×10^{2}	1.48×10^{3}	1.22×10^{3}	8.47×10^{2}	4.14×10^{2}	3.91×10^{2}	3.52×10^{2}	**3.30E+00**
F30	Mean	3.63×10^{5}	7.07×10^{5}	2.53×10^{6}	1.76×10^{5}	4.82×10^{5}	**3.33E+03**	3.41×10^{3}	3.60×10^{3}	3.56×10^{3}
	STD	5.08×10^{5}	3.47×10^{5}	6.38×10^{5}	6.61×10^{4}	3.52×10^{5}	**8.78E+02**	1.46×10^{3}	1.30×10^{3}	1.38×10^{2}
Friedman mean test		5.70	6.80	7.50	5.30	4.50	3.10	3.80	2.6	**1.72**
Rank		7	8	9	6	5	3	4	2	**1**

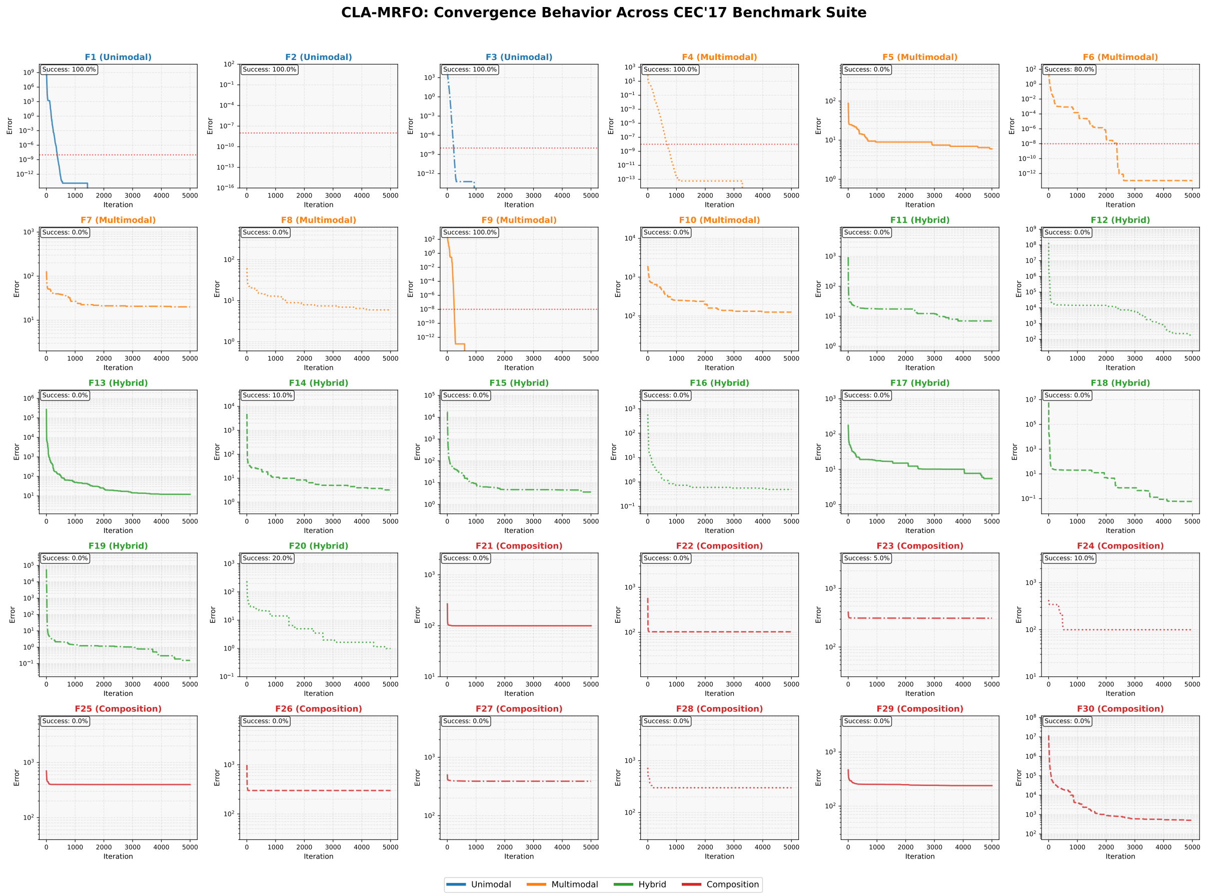

Figure A.1: Convergence behavior of the CLA-MRFO algorithm across the CEC'17 benchmark suite. Each subplot shows the median error (fitness - known bias) over iterations for a single function, illustrating the algorithm's performance across unimodal (F1–F3), multimodal (F4–F10), hybrid (F11–F20), and composition (F21–F30) function types. The red dotted line indicates the success threshold (10^{-8}).
